# Genome-Wide Analysis of the *NF-YB* Gene Family in *Gossypium hirsutum* L. and Characterization of the Role of *GhDNF-YB22* in Embryogenesis

**DOI:** 10.3390/ijms19020483

**Published:** 2018-02-06

**Authors:** Yanli Chen, Zhaoen Yang, Yanqing Xiao, Peng Wang, Ye Wang, Xiaoyang Ge, Chaojun Zhang, Xianlong Zhang, Fuguang Li

**Affiliations:** 1 State Key Laboratory of Cotton Biology, Institute of Cotton Research, Chinese Academy of Agricultural Sciences, Anyang 455000, China; cylxr2012@163.com (Y.C.); yangzhaoen0925@126.com (Z.Y.); 15197272509@163.com (Y.X.); wangpeng19880314@126.com (P.W.); wangye_916@163.com (Y.W.); gexiaoyang613@163.com (X.G.); zcj1999@yeah.net (C.Z.); 2 National Key Laboratory of Crop Genetic Improvement, Huazhong Agricultural University, Wuhan 40070, China; xlzhang@mail.hzau.edu.cn; 3 Xinjiang Research Base, State Key Laboratory of Cotton Biology, Xinjiang Agricultural University, Urumqi 830052, China

**Keywords:** genome-wide analysis, NF-YB transcription factor, *Gossypium hirsutum*, overexpression, embryogenesis

## Abstract

Members of the *NF-YB* transcription factor gene family play important roles in diverse processes related to plant growth and development, such as seed development, drought tolerance, and flowering time. However, the function of *NF-YB* genes in cotton remains unclear. A total of 23, 24, and 50 *NF-YB* genes were identified in *Gossypium arboreum* (*G. arboreum*), *Gossypium raimondii* (*G. raimondii*), and *G. hirsutum*, respectively. A systematic phylogenetic analysis was carried out in *G. arboretum*, *G. raimondii*, *G. hirsutum*, *Arabidopsis thaliana*, cacao, rice and, sorghum, where the 150 *NF-YB* genes were divided into five groups (α–ε). Of these groups, α is the largest clade, and γ contains the LEC1 type NF-YB proteins. Syntenic analyses revealed that paralogues of *NF-YB* genes in *G. hirsutum* exhibited good collinearity. Owing to segmental duplication within the A sub-genome (A_t_) and D sub-genome (D_t_), there was an expanded set of *NF-YB* genes in *G. hirsutum*. Furthermore, we investigated the structures of exons, introns, and conserved motifs of *NF-YB* genes in upland cotton. Most of the *NF-YB* genes had only one exon, and the genes from the same clade exhibited a similar motif pattern. Expression data show that most *NF-YB* genes were expressed ubiquitously, and only a few genes were highly expressed in specific tissues, as confirmed by quantitative real-time PCR (qRT-PCR) analysis. The overexpression of *GhDNF-YB22* gene, predominantly expressed in embryonic tissues, indicates that *GhDNF-YB22* may affect embryogenesis in cotton. This study is the first comprehensive characterization of the *GhNF-YB* gene family in cotton, and showed that *NF-YB* genes could be divided into five clades. The duplication events that occurred over the course of evolution were the major impetus for *NF-YB* gene expansion in upland cotton. Collectively, this work provides insight into the evolution of *NF-YB* in cotton and further our knowledge of this commercially important species.

## 1. Introduction

Nuclear factor Y (NF-Y), also called heme activator protein (HAP) or CCAAT-binding factor (CBF), can be found in almost all eukaryotes. Genes are normally regulated by transcription factors via the specific interactions between the upstream promoter regions and proteins encoded by transcription factors. The CCAAT-box, a common and conserved eukaryotic promoter element, is associated with large range of trans-acting factors, where only the NF-Y is absolutely required for gene regulation [1]. The NF-Y consists of three different subunits: NF-YA (CBF-B or HAP2), NF-YB (CBF-A or HAP3), and NF-YC (CBF-C or HAP5) [2]. All NF-Y subunits contain a highly conserved core region for subunit interactions, which are vital to the function of the transcription factor [3]. The NF-YB subunit includes an amino-terminal A domain, a B domain, and a carboxyl-terminal C domain [4]. Of these, the B domain is the most essential owing to the presence of amino acid residues necessary for its interaction with NF-YA and NF-YC [5]. Moreover, the NF-YB subunit can be divided into two classes in *A*. *thaliana* according to sequence: the LEC1-type and the non-LEC1-type, which differ in the 16 amino acid (aa) residues at equivalent positions in the B domain [6]. The LEC1-type contains LEC1 and LEC1-LIKE (L1L), while the rest belong to the non-LEC1-type [7]. 

Although, NF-YB is generally encoded by only one gene in animals and yeast, there are multiple genes encoding NF-YB in plants [8]. To date, the *NF-YB* gene family has been identified and characterized in several plant species. For example, there are 13 annotated *NF-YB* genes in the model plant *A. thaliana* [9]. As two representative species of monocotyledons, rice and wheat both have 11 *NF-YB* genes [10,11]. Moreover, 14, 32, 7, 18, and 29 *NF-YB* genes have been characterized in canola, soybean, tung tree, grape, and tomato, respectively [12,13,14,15], indicating that the *NF-YB* gene family has been expanded in plants. This expansion suggests that the function of *NF-YB* genes are more complex than previously thought owing to the genetic redundancy and functional divergence of the gene family over the course of evolution.

There is a large body of evidence that *NF-YB* genes have multiple functions. It has been demonstrated that the overexpression of *AtLEC1* (*AtNF-YB9*), a well characterized *NF-YB* gene in *A. thaliana*, in *lec1* mutant and wild-type *A. thaliana* can induce embryo-like structures on the leaves [6]. Moreover, *AtLEC1* has also been reported to be an essential regulator in zygotic embryogenesis, seed maturation, and fatty acid synthesis [16,17]. In contrast to *AtLEC1*, other *NF-YB* genes in *A. thaliana* have been shown to function in drought tolerance, abscisic acid signalling transduction, flowering, and root elongation [18,19,20,21]. Aside from *A. thaliana*, the functional characterization of *NF-YB* genes also have been performed in several other staple crops, and have exhibited varying biological roles. For example, *BnLEC1* and *ZmLEC1* have been reported to increase oil content in seeds [22,23]. Furthermore, *NF-YB* genes have been shown to be involved in the process of chloroplast biogenesis in rice, and fruit ripening in the tomato [10,12,24], while the over-expression of a single *NF-YB* gene in wheat resulted in a 20–30% increase in grain yield [25]. In another study, *VfNF-BY* genes have been shown to play a vital role in pathogen response in the tung tree [4]. Even though *NF-YB* genes have been identified and characterized in dozens of plant species, the members and roles of this gene family in cotton, most notably in upland cotton (*G. hirsutum)*, remain unclear. Thanks to the *Gossypium* sequencing project, many *Gossypium* species have been sequenced, including upland cotton and its two diploid progenitors (https://www.cottongen.org/). The accessibility of these genome sequences allows us to comprehensively identify and characterize *NF-YB* genes in cotton [26,27,28,29]. 

Upland cotton is an economically important crop, which supplies natural and renewable fibre for the textile industry. The aim of the current study was to systematically analyse *NF-YB* genes in *G. hirsutum* (*GhNF-YBs*) using a genome-wide analysis. As a result, 50 members of the *NF-YB* gene family were identified and further characterized to infer the phylogenetic relationships, chromosome locations, gene structures, and conserved motifs of *GhNF-YBs*. In addition, we analysed the expression patterns of *GhNF-YB* genes in different tissues. Lastly, the possible function of *GhDNF-YB22* was characterized by overexpression in cotton. Here, our results will provide a foundation for the future study of *NF-YB* genes in upland cotton and further our understanding of this commercially important species.

## 2. Results

### 2.1. Identification of NF-YB Genes in Cotton

The *A. thaliana* protein sequences of the *NF-YB* gene family were used as queries to search *NF-YB* genes in the *G. arboretum*, *G. raimondii*, *G. hirsutum*, rice, sorghum, and cacao genomes. In total, 23, 40, 52, 16, 18, and 21, respectively, putative *NF-YB* genes were detected. InterProScan 56.0 was used to identify the *NF-YB* genes, where 23, 24, 50, 12, 15, and 13 *NF-YB* genes were successfully identified in the *G. arboretum*, *G. raimondii*, *G. hirsutum*, rice, sorghum, and cacao genomes, respectively (Table S1). The cotton *NF-YB* genes were named based on the distribution locations on the chromosomes (Table S1). We determined that the numbers of gene were very close in the two diploid cotton *G. arboreum* (AA) and *G. raimondii* (DD) species, where the total numbers of genes in the two diploid cottons were slightly smaller than that of the allotetraploid cotton *G. hirsutum*. However, the numbers of *NF-YB* genes in the two diploid cottons were much greater than in rice, sorghum and cacao, indicating that the *NF-YB* gene family has expanded during the evolution of *Gossypium* species. The protein sequence length of *GhDNF-YB16* was 746 amino acid (aa), while the length of the orthologue *GhANF-YB16* was 173 aa. To further verify the differences in sequences between *GhDNF-YB16* and *GhANF-YB16*, we designed primers (Table S2) for *GhANF-YB16* and cloned it from upland cotton. The results showed that the nucleic acid sequence of *GhANF-YB16* was shorter than that of *GhDNF-YB16* owing to transcription termination. The length of NF-YB protein sequences ranged from 90 to 318 aa in our study.

### 2.2. Phylogenetic Analysis of the NF-YB Gene Family

**Figure 1:**
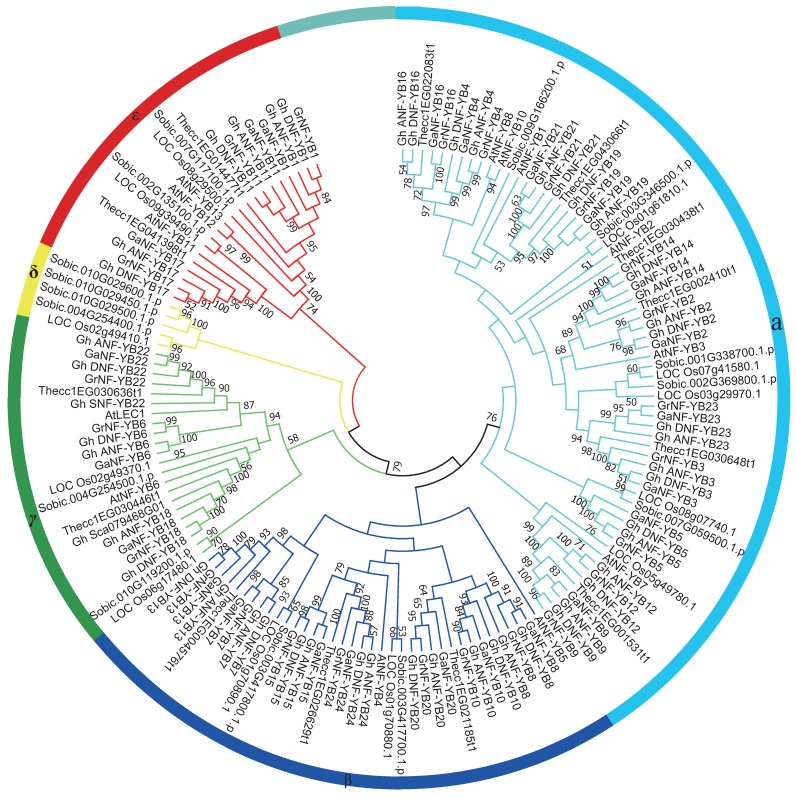
Phylogenetic relationships of *NF-YB* gene family. The analysis included full-length protein sequences from *Gossypium hirsutum*, *Gossypium arboretum*, *Gossypium raimondii*, *Arabidopsis*, *Oryza sativa*, *Sorghum bicolor*, and *Theobroma cacao*. Using MEGA software, the phylogenetic tree was constructed with 1000 bootstrap replicates using the neighbour-joining method, where only bootstrap values >50% are shown. A total of 150 NF-YB proteins were divided into five branches corresponding to subunit type, and are indicated by different colours.

To better understand the evolutionary relationships of *NF-YB* gene, a neighbour-joining (NJ) phylogenetic tree was constructed using the *NF-YB* genes from *G. hirsutum*, *G. arboretum*, *G. raimondii*, *A. thaliana*, rice, sorghum, and cacao. As shown in Figure 1, the *NF-YB* genes were naturally divided into five clades, designated as α, β, γ, δ, and ε. The α clade was the largest group, containing 65 *NF-YB* genes, whereas the δ clade was the smallest, consisting of only five members, indicating that *NF-YB* genes were distributed unevenly in the different clades. The α, β, γ, and ε clades consisted of genes both from dicot and monocot species, while the δ clade only contained genes from monocot species, including four *NF-YB* genes from sorghum and one from rice. According to the presence of the typical LEC1 motif—consisting of 16 shared residues in the B domain—NF-YB proteins can be classified as either LEC1 type or non-LEC1 proteins. We found that only the members of the γ clade can be classified as LEC1 type proteins. GhA/DNF-YB6, GhA/DNF-YB18, and GhA/DNF-YB22—typical LEC1-type proteins—share a common ancestor with AtLEC1 and AtLEC1-like proteins (Figure S1), and were determined to be important candidate genes for embryogenesis in cotton. Notably, nearly all the orthologous genes from the two monocot species (sorghum and rice) tended to form orthologous gene pairs at the end of branches in the phylogenetic tree, where *NF-YB* genes from dicots (cotton, cacao, and *Arabidopsis*) tended to cluster together, indicating that the main function of these members of the gene family diverged prior to the divergence of dicots and monocots. As reported by Wang et al. [26], cotton has been experienced a recent duplication event whereas cacao did not, in agreement with our findings that, in most cases, each cacao gene corresponds to two orthologues in diploid cotton. For example, in the ε clade, *cc1EG014477t1* corresponded to two orthologues in both *G. arboreum* and *G. raimondii*. 

### 2.3. Chromosomal Distribution and Synteny Analysis of GhNF-YB Genes

**Figure 2:**
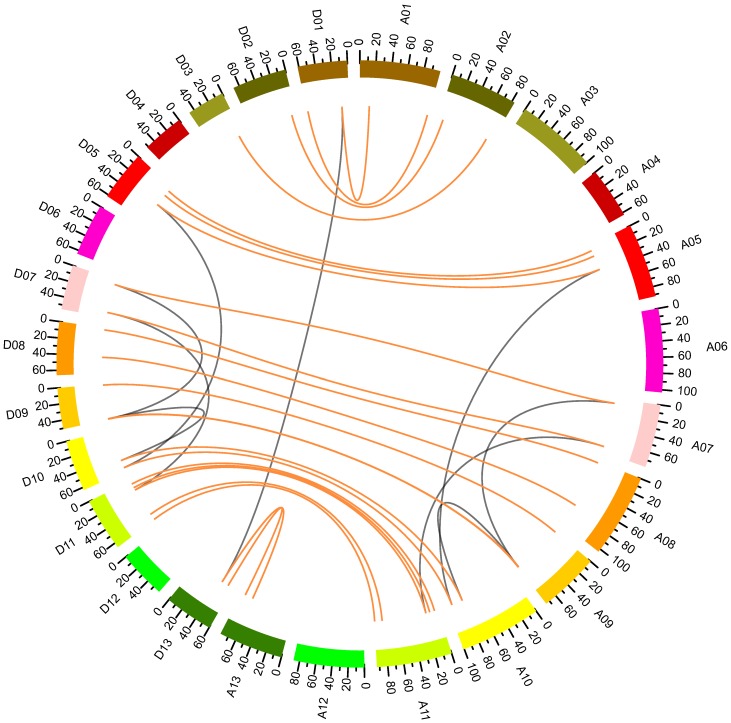
Collinearity analyses of *Gossypium hirsutum NF-YB* genes. A01–13 and D01–13 represent chromosomes from the A and D sub-genomes, respectively. The red lines link two genes that were identified to be homologous chromosome pairs from the A_t_ and D_t_ sub-genomes. The grey lines link gene pairs formed by segmental duplication within the A_t_ and D_t_ sub-genomes.

A total of 50 *NF-YB* genes were detected in *G. hirsutum* and were unevenly distributed on chromosomes, where 48 of the genes detected were located on nine A_t_ chromosomes (A1, A2, A5, A7, A8, A9, A10, A11, and A13) and ten D_t_ chromosomes (D1, D2, D3, D5, D7, D8, D9, D10, D11 and D13) (Figure 2 and Figure S2). The remaining two genes (*GhSNF-YB18*, *GhSNF-YB22*) were distributed on two unoriented scaffolds. The total number of *NF-YB* genes mapped within A_t_ sub-genomes was equal to that of the D_t_ sub-genomes. We found that the distribution of genes was uneven within each chromosome, and most of the orthologues from the A_t_ and D_t_ sub-genomes were located on homologous chromosomes. Nine chromosomes contained two *NF-YB* genes, six chromosomes contained three genes, and two chromosomes contained five genes (Figure 2 and Figure S2). 

*Gossypium hirsutum*, as the typical allotetraploid species, was derived from the hybridization of two diploid species resembling the ancestors of *G. arboretum* and *G. raimondii*, where the resulting chromosome was doubled [30]. Tandem duplication, segmental duplication, and whole-genome duplication are the main impetus for gene family expansion [31]. As shown in Figure 2, the orthologues maintained good collinearity between the A_t_ and D_t_ sub-genomes. A segmental duplication analysis showed that nine pairs of genes may have been derived from segmental duplication events (Table S3). Eight genes formed four pairs of duplicated genes in the D_t_ sub-genome, while their orthologues in the A_t_ subgenome also formed four pairs of duplicated genes accordingly, indicating that the duplication events happened prior to the doubling of the upland cotton chromosome. The results of our duplication analysis were consistent with those of the phylogenetic analysis, as the duplication pairs clustered closely to each other in the phylogenetic tree (Figure 1 and Figure 2). 

Over the course of evolutionary history, duplicated genes have three potential evolutionary fates: non-functionalisation, neo-functionalisation, and sub-functionalisation [32]. In comparing the non-synonymous (Ka) and synonymous substitution (Ks) rates of substitution (Ka/Ks), one could infer the magnitude of selective constraint and positive selection. Generally, Ka/Ks > 1, Ka/Ks = 1, and Ka/Ks < 1 indicate positive selection, neutral evolution, and purifying selection, respectively. In the present study, the Ka, Ks, and Ka/Ks of *NF-YB* homologous gene pairs were estimated in *G. hirsutum* (Table 1). We found that the Ka/Ks ratios of *NF-YB* gene homologous pairs were less than 0.5, and that the ratios of three of these homologous pairs were smaller than 0.1, suggesting that *NF-YB* genes have undergone purifying selection after segmental and whole genome duplications.

Transposable elements (TEs) compose a major fraction of eukaryotic genomes, especially in plants, mainly in retrotransposons and DNA transposons, which move around the genome [33]. Transposable elements are expressed and mobilized in order to respond to specific stimuli [34]. To investigate whether TEs played roles in expansion of the NF-YB protein family, TEs close to the *NF-YB* genes were identified in the present study (Table 2). Only three retroelements—L1 (1) and Copia (2)—were found in the 2000 bp region upstream and downstream of the genes (Table S4). When the scanning region was broadened to 10,000 bp, fifty-four TEs were identified. Of these, only one could be classified as a DNA transposon, while the rest of them were retroelements (i.e., L1 [10], copia [33], and gypsy [10]) (Table S5). Upon further investigation, we found that one L1 was located upstream of *GhDNF-YB1*, and two Copia were located in the gene region of *GhDNF-YB2*, within the 2000 bp region. Moreover, within 10,000 bp region, one DNA/hAT-Ac was located downstream of *GhDNF-YB6*; two L1 elements were located upstream of *GhANF-YB6* and downstream of *GhDNF-YB3* and *GhANF-YB10*; one L1 element was located downstream *of GhANF-YB3* and upstream of *GhANF-YB21*, *GhDNF-YB21*, and *GhDNF-YB1*; seven Copia were located downstream of *GhDNF-YB18*; five Copia were located upstream of *GhDNF-YB10* and *GhDNF-YB14*; four Copia elements were located downstream of *GhANF-YB19*; two Copia elements were located within the gene region of *GhANF-YB2* and upstream of *GhDNF-YB3*, *GhANF-YB3*, and *GhDNF-YB21*; one Copia element was located downstream of *GhDNF-YB15* and *GhDNF-YB20* and upstream of *GhANF-YB13* and *GhANF-YB1*; three gypsy elements were located upstream of *GhDNF-YB24* and *GhANF-YB23*; and one gypsy element was located downstream of *GhANF-YB14*, *GhANF-YB2* and upstream of *GhDNF-YB14* and *GhDNF-YB1.* We noted that most of the TEs were located in the vicinity of duplicated genes, suggesting that TEs contributed to the expansion of the *NF-YB* gene family. The numbers of simple repeat sequences were more abundant than those of TEs, and their lengths were variable, which could play important roles in functional divergence after duplication.

### 2.4. Gene Structure and Analysis of Conserved Motifs

**Figure 3:**
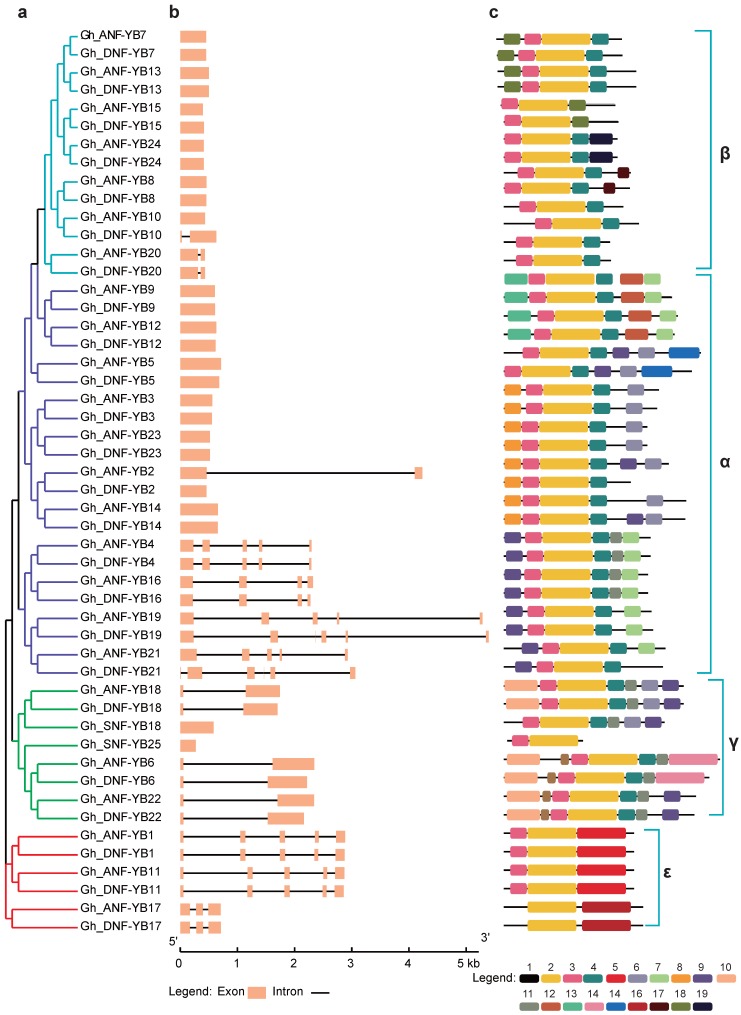
Phylogenetic relationships, exon-intron structures, and conserved motifs of *NF-YB* genes in *Gossypium hirsutum*. (**a**) An unrooted tree was constructed in MEGA using the neighbour-joining method, while the four subfamilies are indicated by different colours. (**b**) The pink boxes and black lines indicate exons and introns, respectively. (**c**) The distribution of conserved motifs in GhNF-YB family, where motif 2 represents the B domain.

To comprehensively study the phylogenetic relationships between the *NF-YB* genes, we performed analyses of gene structure and conserved motifs. As shown in Figure 3a, the *NF-YB* genes were classified into five clades that were consistent with the phylogenetic relationships illustrated in Figure 1. To elucidate the gene structure of the *GhNF-YB* family, we compared coding sequences to their corresponding genomic sequences to determine positions of the exons and introns position the genomic sequences. As shown in Figure 3b, the numbers of exons ranged from one to six, where genes with one exon accounted for 60% of the total *NF-YB* genes, most of which were from the α and β clades. In analysing the conserved motifs in the *GhNF-Y B* genes using MEME, we found that all 50 NF-YB proteins shared motif 2 (yellow box) (Figure 3c), which was contained within the B domain. In addition, most of the NF-YB proteins contained similar motifs. For instance, motifs 3 and 4 were widely distributed. We also found that *NF-YB* genes with close phylogenetic relationships exhibited similar arrangements of motifs. We also identified the pattern of amino acid residues conservation in the domains of GhNF-YBs (Figure S3). 

### 2.5. Analyses of Tissue-Specific Expression Patterns of 50 G. hirsutum NF-YB Genes

**Figure 4:**
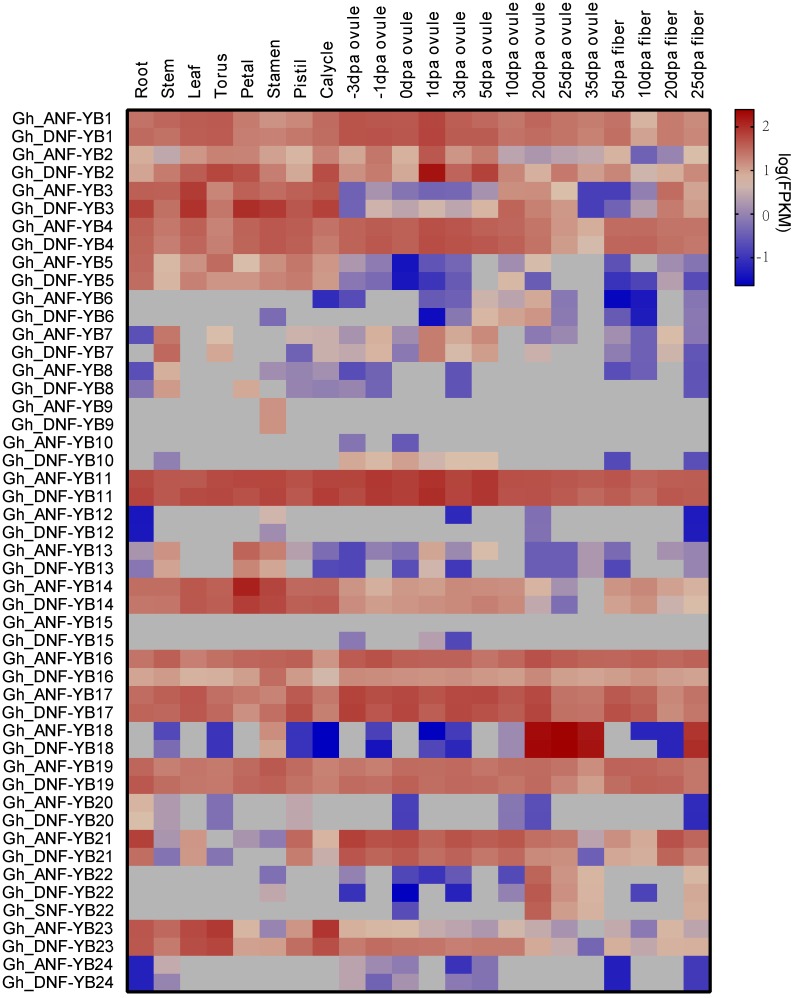
Gene expression patterns of *NF-YB* genes in a variety of upland cotton tissues. The raw data for RNA-Seq were downloaded from NCBI and analysed using Tophat and Cufflinks [35]. Gene expression levels are depicted with different colour on the scale. Blue and red represent low and high expression, respectively.

To assess the expression patterns of *GhNF-YB* genes, RNA-seq data were downloaded from NCBI and analysed. Gene expression patterns of *GhNF-YB* genes were analysed in a variety of tissues in *G. hirsutum*, including vegetative tissues (root, stem and leaf), reproductive tissues (some parts of the floral organ), and fibre (5, 10, 20, and 25 d post-anthesis). As shown in Figure 4, we found that some *NF-YB* genes were widely expressed in all of the aforementioned tissues, indicating that these genes have important biological functions during plant development. For example, *GhA/DNF-YB4*, *GhA/DNF-YB16*, and *GhA/DNF-YB19* exhibited very high levels of expression in vegetative tissues, reproductive tissues, and fibre. In contrast, other genes exhibited much different expression patterns. Specifically, *GhA/DNF-YB9* was expressed in the stamen, while *GhA/DNF-YB18* and *GhA/DNF-YB22* were preferentially expressed in 20, 25, and 35 days post-anthesis (DPA) ovules and 25 DPA fibres. *GhA/DNF-YB1*, *GhA/DNF-YB11*, and *GhA/DNF-YB17* not only exhibited phylogenetic relationships (Figure 1 and Figure 3), but also similar expression patterns. An additional investigation revealed that the syntenic duplicates, with the exception of *GhA/DNF-YB11/1*, were divergent in expression patterns, indicating sub-functionalisation. 

**Figure 5:**
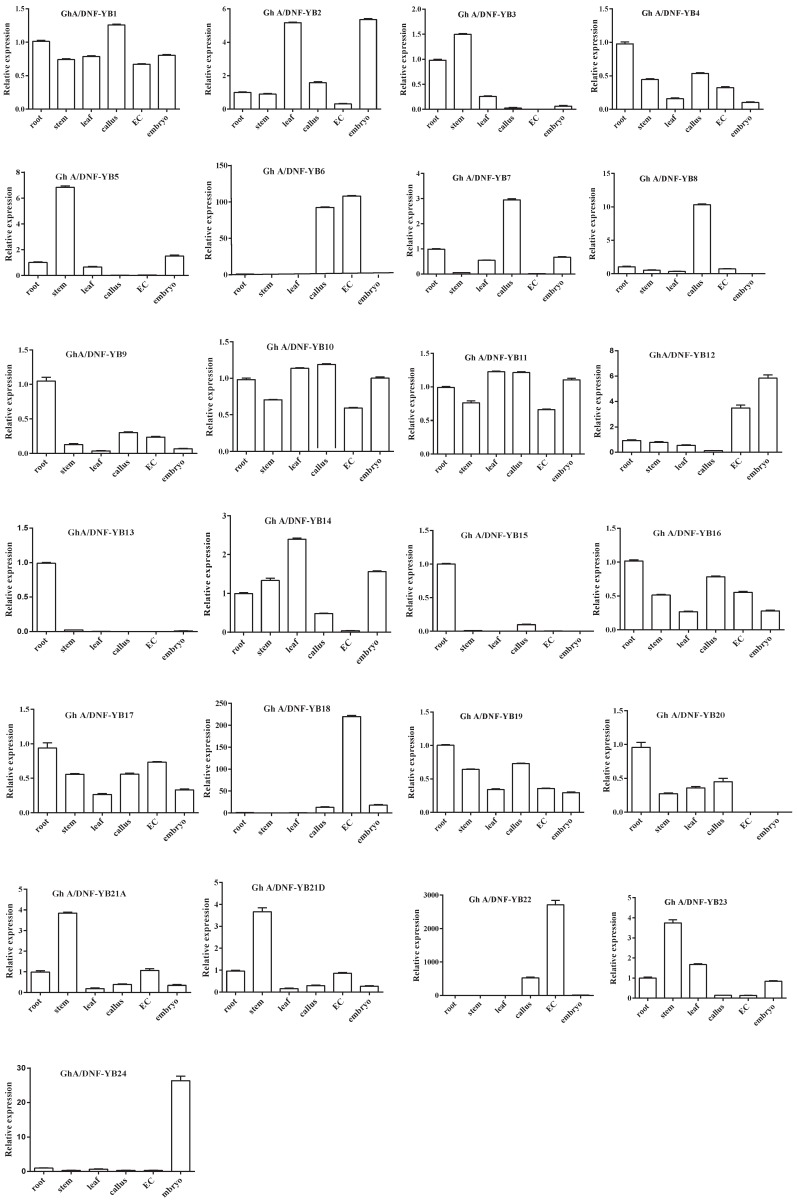
Expression levels of *NF-YB* genes in different tissues, as determined by qRT-PCR. Error bars represent the standard deviations of three independent experiments.

To validate the expression levels of *GhNF-YBs*, qRT-PCR was used to test gene expression in the root, stem, leaf, callus, embryogenic callus, and embryo. The results of the qRT-PCR were in agreement with expression patterns observed in the analysis of the RNA-seq data (Figure 5). For example, *GhA/DNF-YB1*, *GhA/D NF-YB11*, and *GhA/DNF-YB17* were expressed in all tissues selected, while *GhA/DNF-YB6*, *GhA/DNF-YB18*, and *GhA/DNF-YB22* exhibited very high expression levels only in several selected tissues (callus and embryogenic callus). In contrast, *GhA/DNF-YB9*, *GhA/DNF-YB12*, *GhA/DNF-YB13*, and *GhA/DNF-YB24* were very lowly expressed in any of the tissues assayed.

### 2.6. Overexpression of GhDNF-YB22 in Cotton Affects Embryogenesis

**Figure 6:**
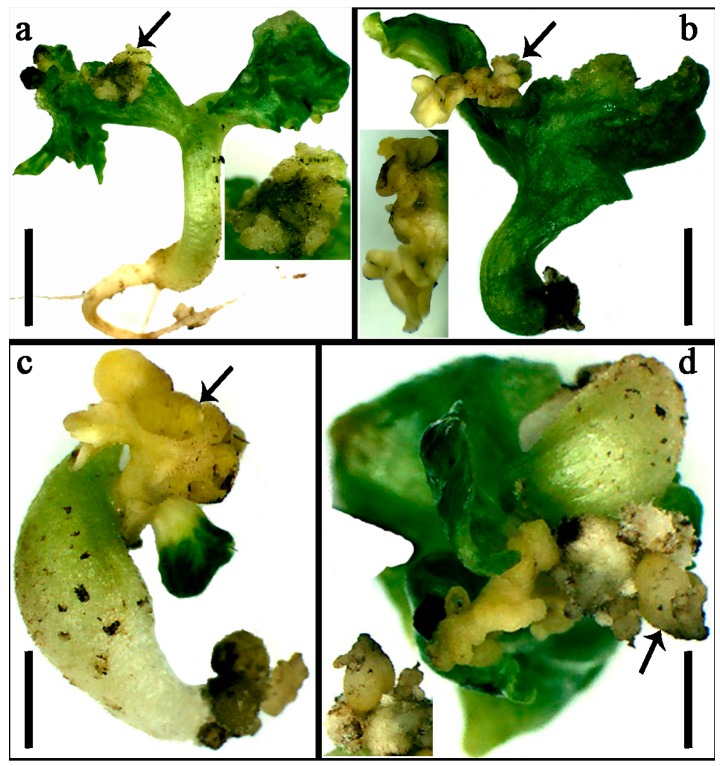
Phenotypes of transgenic cotton seedlings ectopically expressing *GhDNF-YB22*: (**a**) seedlings produced a callus-like structure; (**b**) seedling produced embryo-like organs; (**c**) embryo-like organs were substituted for leaf growth; and (**d**) embryo-like structures developed from the callus. Bars: 0.5 mm (**a**,**b**); and 0.1 mm (**c**,**d**).

*GhA/DNF-YB6*, *GhA/DNF-YB18*, *GhA/DNF-YB22*, *AtLEC1*, and *AtNF-YB6* were clustered in the γ clade (Figure 1). In *A. thaliana*, LEC1 is a main regulator of embryogenesis [36]. To characterize the function of the *GhNF-YB* gene, *GhDNF-YB22*, which is highly homologous to *AtLEC1*, *GhDNF-YB22* was transformed into cotton under the control of the CaMV35 promoter. After performing the *Agrobacterium*-mediated transformation of cotton hypocotyl, hypocotyl somatic cells underwent dedifferentiation and redifferentiation, formed the callus and embryogenic callus, then produced somatic embryo, and lastly developed into new plants. Over the course of these processes, we found that transgenic seedlings exhibited a set of morphological phenotypes. Callus-like structures formed on the leaf-like organ surfaces of seedlings (Figure 6a), while some embryo-like structures developed from the callus-like structures (Figure 6d). Remarkably, some embryo-like structures emerged on the margins of leaf-like organs (Figure 6b), or substituted for growth of leaves (Figure 6c). The transgenic lines of *GhDNF-YB22* were determined by kanamycin selection and qRT-PCR test (Figure S4). These resulting morphological phenotypes indicate that *GhDNF-YB22* plays an important role in embryogenesis.

## 3. Discussion

The *NF-YB* gene family had been previously analysed in several plant species, including *A. thaliana*, rice, wheat, tung tree, soybean, canola, grape, and tomato. However, a genome-wide identification and characterization of *NF-YB* genes has not been reported in *G. hirsutum*, an allotetraploid species. In the present study, we conducted an integrated investigation of the *GhNF-YBs*, consisting of phylogenetic analyses, an investigation of expression patterns, and transgenic verification.

### 3.1. Variation in the NF-YB Gene Family in G. hirsutum

In the present study, nearly all of the orthologues from two monocot species (sorghum and rice) and three dicots (cotton, cacao, and *Arabidopsis*) tended to cluster together, indicating that the main functions of the *NF-YB* gene family diverged prior to the divergence of dicots and monocots.

The allotetraploid cotton *G. hirsutum* was derived from the hybridization of an A-genome species resembling *G. arboreum* and a D-genome species resembling *G. raimondii* [26], followed by a chromosome doubling event. Because of the whole genome duplication, the upland cotton experienced polyploidisation, which results in an extensive reshuffling of the entire genome [37]. At present, there is much evidence to support the notion that the gain and loss of genes or the expansion or contraction of gene families is common following polyploidisation [38,39]. Thus, the expansion of the *GhNF-YB* gene family also could be an indication that *GhNF-YB* genes play roles in additional biological processes or have novel functions, in agreement with the allotetraploid nature of *G. hirsutum* [40,41,42]. An analysis of collinearity showed that orthologous genes maintained good collinearity between the A_t_ and D_t_ sub-genomes, while segmental duplication analysis showed that nine pairs of genes may be derived from segmental duplication (Figure 2). These results suggest that segmental duplication also played an important role in the expansion of the *NF-YB* gene family.

In analysing gene structure, we found that many *NF-YB* genes in *G. hirsutum* had only one exon with no introns (Figure 3), which is consistent with findings in *Arabidopsis* and *Brassica napus* L. [13]. Previous studies have postulated that an intron-rich gene would lose multiple introns simultaneously by retrotransposition, thereby producing intron-less ancestral genes [43]. Thus, some *NF-YB* genes in *G. hirsutum* may experience the loss of multiple introns during gene family diversification. Genome-wide analyses have shown that the loss and gain of introns has been extensive during the process of eukaryotic diversification [44,45].

### 3.2. Expression Patterns of NF-YB Genes in G. hirsutum

Previous studies have reported that *NF-YB* genes play important roles in plant developmental processes (e.g., in late embryogenesis, flowering time, drought tolerance, etc.) [46]. In the present study, we identified the tissue-specific expression patterns of *GhNF-YB* genes in a variety of tissues, where the results show that most of the *NF-YB* genes are expressed ubiquitously, with the exception of a few genes that are expressed in specific tissues (Figure 5). This observation was consistent with previous studies [10], suggesting that *NF-YB* genes are polyfunctional and are involved in a wide range of biological processes [47]. 

In phylogenetic analysis, *GhNF-YB* genes were divided into five clades with several *G. hirsutum*- and *A. thaliana*-specific *NF-YB* genes, with the exception of the δ clade. Of these, *NF-YB1*, *NF-YB2*, *NF-YB3*, *NF-YB*6, and *NF-YB9* have been extensively studied in *A. thaliana*. Previous studies revealed that *NF-YB1* not only regulated drought tolerance [18], but also interacted with CO (CONSTANS) to affect the transcript levels of two key integrators (FT: FLOWERING LOCUS T and SOC1: SUPPRESSOR OF OVEREXPRESSION OF CO1) in the flowering pathway, and therefore adjusted the flowering time [48]. Interestingly, *GhA/DNF-YB21* and *GhA/DNF-YB19* clustered with *AtNF-YB1*, where *GhA/DNF-YB19* was expressed in all selected tissues, while *GhA/DNF-YB21* was mainly expressed in reproductive tissues. These observations indicate that *GhA/DNF-YB21* and *GhA/DNF-YB19* may have similar functions as *AtNF-YB1*. Moreover, *GhA/DNF-YB2*, *GhA/DNF-YB3*, *GhA/DNF-YB14*, and *GhA/D NF-YB23* were observed to cluster with *AtNF-YB2* and *AtNF-YB3*, which have been reported to regulate the photoperiod-dependent flowering time [20]. In barley, *HvNF-YB3* and *HvNF-YB1* clustered with *AtNF-YB2* and *AtNF-YB3*, and have been shown to greatly promote early flowering [49]. *NF-YB9/LEC1* was the first *NF-YB* gene identified and studied in *A. thaliana*, and has been shown to be required for the maintenance embryonic of cell fate, where the ectopic expression of *LEC1* can induce somatic embryos from vegetative cells [36]. In addition, *LEC1* has also been shown to play an essential role in embryogenesis and seed maturation [6,50]. *LEC1* and *LEC1-LIKE* (*NF-YB6*) regulated embryo development by activating the expression of genes required for embryogenesis and cellular differentiation [7,36]. In the present study, *GhA/DNF-YB*6 and *GhA/DNF-YB22* were grouped with *AtLEC1*, while *GhA/DNF-YB18* was grouped with *AtLEC1-LIKE*. Furthermore, *GhA/DNF-YB6*, *GhA/DNF-YB18*, and *GhA/DNF-YB22* were all highly expressed in the callus and embryogenic callus as evidenced by qRT-PCR. Thus, these three paralogue pairs may be involved in regulating embryonic development. 

### 3.3. Role of GhDNF-YB22 in Embryogenesis

*LEC1* has been shown to function in different aspects of embryogenesis, such as embryonic development, the induction of embryogenesis at morphogenesis and maturation phases, the induction of embryonic programs in vegetative cells, and the identification of cotyledons [36,51]. The function of LEC1 is conserved in seed development by regulating distinct genes at different developmental stages in *Arabidopsis* and soybean [52]. In addition, vegetative or reproductive cells could change their fate and exhibit somatic embryo development via the ectopic expression of LEC [53]. Here, *GhDNF-YB22* was ectopically expressed in upland cotton, whereupon callus- and embryo-like structures emerged on the leaf-like organs as a result (Figure 6). This in agreement with 35S/LEC1 seedlings, which produced multiple embryo-like structures on the leaves of *Arabidopsis* [36]. This indicates that *GhDNF-YB22* is functionally similar to *LEC1*, which promotes the transcription of genes required for embryo morphogenesis. Furthermore, *GhA/DNF-YB6*, *GhA/DNF-YB18* and *GhA/DNF-YB22* in γ clade have been revealed conservative exon-intron structures and expression patterns (Figure 3 and Figure 4). These indicate that *NF-YB* genes in γ clade may have similar biological function in embryogenesis.

## 4. Materials and Methods

### 4.1. Identification of the NF-YB Gene Family

The protein sequences of NF-YB in *A. thaliana* (http://www.arabidopsis.org) were used as queries to search the sequences of *G. arboretum*, *G. raimondii*, *G. hirsutum*, rice, sorghum, and cacao in blastp. Cotton sequences—including *G. arboretum*, *G. raimondii*, and *G. hirsutum*—were downloaded from COTTONGEN (http://www.cottongen.org), while the other aforementioned species here were obtained from phytozome (https://phytozome.jpi.doe.gov/pz/portal.html). In addition, InterProScan 56.0 (http://www.ebi.ac.uk/inerpro/) was used to identify the *NF-YB* gene family numbers.

### 4.2. Phylogenetic Analyses

NF-YB proteins from seven plant species (*A. thaliana*, *O. sativa*, *G. arboreum*, *G. raimondii*, *G. hirsutum*, *T. cacao*, and *S. bicolor*) were used in a multiple alignment in CLUSTAL-X [54]. Subsequently, a phylogenetic tree based on NF-YB protein sequences was constructed via the neighbour-joining method using MEGA 7.0 (http://www.megasoftware.net/) [55]. To establish the reliability of the phylogenetic analysis, the *p*-distance method with 1000 bootstrap samples was used with pairwise deletion and a Poisson correction.

### 4.3. Chromosome Locations and Collinearity Analyses

The loci of *NF-YB* genes were obtained from the genome annotation data. Mapchart was applied to map the chromosome locations [30]. The basic local alignment search tool (BLAST) [56] was used to retrieve the GhNF-YB protein sequences from a local database. Next, these sequences were analysed to identify the collinearity blocks against the whole genome using MCSCAN (http://chibba.agtec.uga.edu/duplication/mcscan/) [30], while CIRCOS software (http://circos.ca/) was used to draw the collinearity map [57]. 

### 4.4. Estimating Ka/Ks Rates

Using Clustal X 2.0 (ftp://ftp.ebi.ac.uk/pub/software/clustalw2/) [54], the amino acid sequences from duplicated pairs were aligned and the aligned sequences converted to cDNA using PAL2NAL (http://www.bork.embl.de/pal2nal/). Lastly, the synonymous (Ks) and nonsynonymous (Ka) substitution rates were estimated using the CODEML program of PAML (http://abacus.gene.ucl.ac.uk/software/paml.html) [58].

### 4.5. Analysis of Transposable Elements

To study the function of transposable elements (TEs) in the NF-YB family, we identified and analysed the different types of TEs in the 2000 and 10,000 bp upstream and downstream regions of the gene. PILER-DF, RepeatModeler, and LTR_FINDER [59,60] were used to predict TEs. Using RepbaseTE (http://www.girinst.org/repbase/), the TEs were identified at the DNA level with RepeatMasker (http://repeatmasker.org/).

### 4.6. Gene Structure and Conserved Motifs Analysis

The Gene Structure Display Server (GSDS) (http://gsds.cbi.pku.edu.cn/) was employed to analyse the exon-intron structure of *GhNF-YB* genes using cDNAs and corresponding genomic sequences. The online program Multiple Em for Motif Elicitation (MEME) (http://meme-suite.org/tools/meme) was chosen to identify the conserve motifs in all GhNF-YB proteins according to the following parameters: the optimum width of motifs ranged from 6 to 200 aa, and the maximum number of motifs to find was defined at 20. The annotations of the identified motifs were completed by the program of InterProScan 56.0 (http://www.ebi.ac.uk/interpro/). 

### 4.7. Gene Expression Heat Map

To measure the expression levels of *NF-YB* family genes, raw data from the RNA-sequencing of various tissues (i.e., root, stem, leaf, torus, petal, stamen, pistil, calycle, ovule and fibre) in *G. hirsutum* cultivar TM-1 was downloaded from NCBI (https://www.ncbi.nlm.nih.gov/bioproject/PRJNA248163/). Then the data were normalized to calculate the expression levels. Subsequently, Genesis software (http://www.gsoft.com.au/) was used to draw the heat map [61]. 

### 4.8. RNA Isolation and qRT-PCR Verification

The seeds of *G. hirsutum* cultivar CCRI24 were grown in a field in Anyang, China. Root, stem, and leaf tissue were sampled and frozen in liquid nitrogen, and subsequently stored at −80 °C. In addition, the seeds of CCRI24 were rinsed with 70% ethanol for 1 min, washed three times with sterile distilled water, and soaked for 24 h in 30% H_2_O_2_. The sterilized seeds were germinated on MS medium (PH: 5.8–6.0) for 7 days, and the hypocotyls of aseptic seedlings were cut into approximately 5 mm sections and used as explants. The explants were cultured using different media for the callus, embryogenic callus, and somatic embryos according to previously published methods [62]. The callus, embryogenic callus, and somatic embryos were sampled and frozen at −80% until RNA extraction. Total RNA was extracted from prepared samples using the RNAprep Pure Plant Kit (Tiangen, Beijing, China). The PrimeScript^®^ RT reagent kit (Takara, Dalian, China) was used to synthesize the first strand cDNA using approximately 2 μg of RNA. Gene-specific primers for qRT-PCR were designed using DNAMAN 7.0 (Table S2). The *histone 3* gene in *G. hirsutum* (GenBank accession no.AF024716) was used as an internal control [63,64]. PCR amplifications were performed using SYBR Premix Ex Taq (Takara), according to previously published methods [65]. For each analysis, qRT-PCR assays had three biological replicates, each consisting of three technical replicates. Error bars were standard error of three technical replications. The relative expression levels of *GhNF-YB* genes were calculated by the 2^−ΔΔ*C*t^ method [66].

### 4.9. Gene Cloning and Transformation into Cotton

The mixed cDNA of root, stem, leaf, callus, and embryogenic callus tissues from CCRI24 was synthesized as a template to amplify genes based on gene-specific primers. The complete protein-coding region was cloned into the pCAMBIA2301 vector with the cauliflower mosaic virus 35S (CaMV35) promoter, and the constructed vector was transferred into *Agrobacterium tumefaciens* strain LBA4404 in the subsequent step. Finally, Hypocotyl explants from CCRI24 were transformed using *A. tumefaciens*-mediated transformation according to previously published methods [67,68]. 

## 5. Conclusions

Although the function of some *NF-YB* genes has been demonstrated clearly in several plant species, especially in Arabidopsis, their roles in *G. hirsutum* are still elusive. In the current study, we performed a genome-wide analysis of the *NF-YB* gene family in *G. hirsutum*, including investigated the evolutionary relationships, gene structure and expression patterns. Fifty *NF-YB* genes are identified, and whole genome and segmental duplication might be the major ways for the expansion of the NF-YB family in upland cotton. Furthermore, the duplicated genes showed different expression patterns, indicating that the duplicated genes probably have experienced functional divergence. Our results will provide a foundation for further study of NF-YB gene family in upland cotton.

## Figures and Tables

**Table 1:** Comparative analysis of Ka, Ks, and Ka/Ks values for homologous pairs in *Gossypium hirsutum*.

Homologous Pairs		Ka	Ks	Ka/Ks
*Gh_ANF-YB21*	*Gh_ANF-YB19*	0.071	0.376	0.189
*Gh_ANF-YB11*	*Gh_ANF-YB1*	0.025	0.563	0.044
*Gh_ANF-YB14*	*Gh_ANF-YB2*	0.061	0.621	0.098
*Gh_ANF-YB20*	*Gh_ANF-YB10*	0.203	1.113	0.182
*Gh_DNF-YB23*	*Gh_DNF-YB3*	0.067	0.486	0.139
*Gh_DNF-YB21*	*Gh_DNF-YB19*	0.183	0.474	0.386
*Gh_DNF-YB11*	*Gh_DNF-YB1*	0.025	0.481	0.052
*Gh_DNF-YB14*	*Gh_DNF-YB2*	0.083	0.500	0.165
*Gh_DNF-YB20*	*Gh_DNF-YB10*	0.245	0.846	0.289

**Table 2:** Transposable elements in the vicinity of the *NF-YB* gene locus.

Type	Elements	Number of Elements	Length Occupied (bp)	Percentage of Sequence (%)	Number of Elements	Length Occupied (bp)	Percentage of Sequence (%)
		10,000 bp region			2000 bp region		
DNA transposons		1	91	0.10	0	0	0
	CMC-EnSpm	0	0	0	0	0	0
	MULE-MuDR	0	0	0	0	0	0
	PIF-Harbinger	0	0	0	0	0	0
	TcMar-Pogo	0	0	0	0	0	0
	hAT	0	0	0	0	0	0
	hAT-Ac	1	91	0.10	0	0	0
	hAT-Charlie	0	0	0	0	0	0
	hAT-Tag1	0	0	0	0	0	0
	hAT-Tip100	0	0	0	0	0	0
Retroelements		53	17,673	18.90	3	1038	7.43
	LINE:	10	2923	3.13	1	91	0.65
	L1	10	2923	3.13	1	91	0.65
	LTR:	43	14,750	15.78	2	947	6.78
	Caulimovirus	0	0	0	0	0	0
	Copia	33	12,359	13.22	2	947	6.7
	Gypsy	10	2391	2.56	0	0	0
	RC:	0	0	0	0	0	0
	Helitron	0	0	0	0	0	0
DNA		1	72	0.08	0	0	0
Low_complexity		166	9514	10.18	47	2479	17.74
Simple_repeat		586	25176	26.93	221	8121	58.12
Unspecified		151	49452	52.90	16	3681	26.35
tRNA		1	30	0.03	0	0	0

## Supplementary Materials

Supplementary Materials can be found at http://www.mdpi.com/1422-0067/19/2/483/s1.
