# Supplementary material for: Genome-Wide Analysis of the NF-YB Gene Family in Gossypium hirsutum L. and Characterization of the Role of GhDNF-YB22 in Embryogenesis
Source: Int J Mol Sci. 2018 Feb 6;19(2):483. doi: 10.3390/ijms19020483 (PMC5855705; doi:10.3390/ijms19020483)
Supplement: Supplementary file 1 [file ijms-19-00483-s001.zip › ijms-265162-supplementary/supplementary materials/Supplementary Figure S1.pdf]

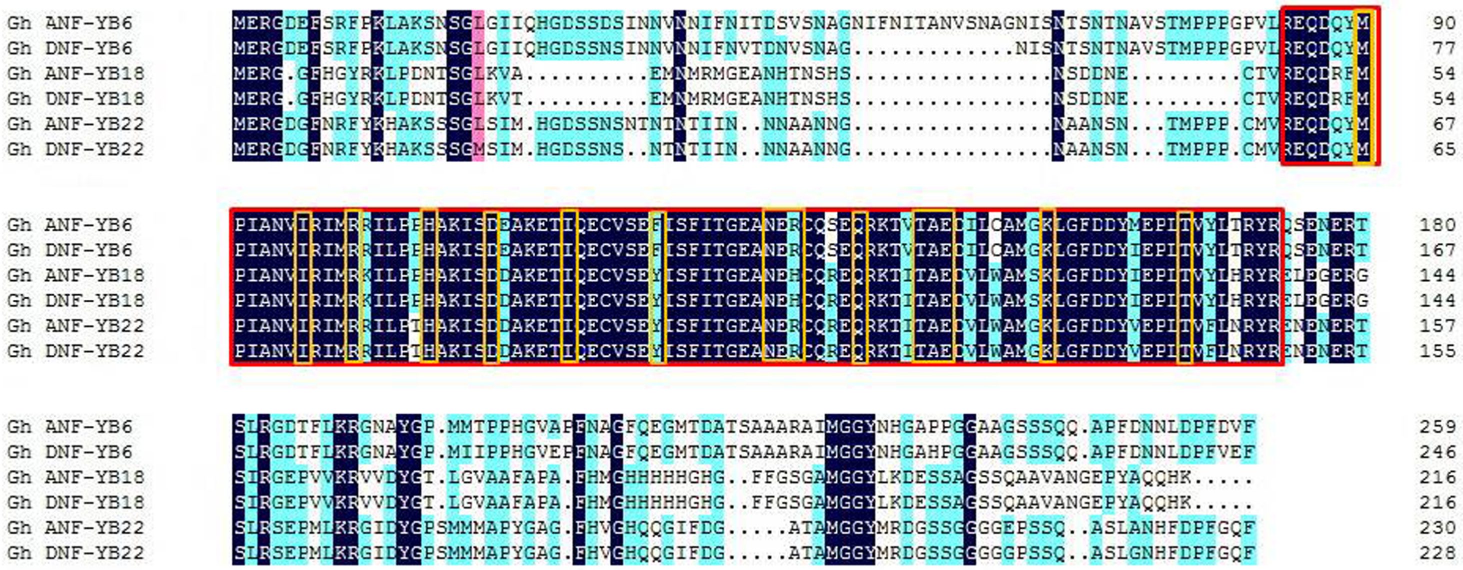

Gh ANF-YB6 MERGDEFSRFPKLAKSNSGLGIIQHGDSSDSINNVNNIFNITDSVSNAGNIFNITANVSNAGNISNTSNTNAVSTMPPPGPVLREQDQYM 90
Gh DNF-YB6 MERGDEFSRFPKLAKSNSGLGIIQHGDSSNSINNVNNIFNVTDNVSNAG.............NISNTSNTNAVSTMPPPGPVLREQDQYM 77
Gh ANF-YB18 MERG.GFHGYRKLPDNTSGLKVA..........EMNMRMGEANHTNSHS...............NSDDNE.........CTVREQDRFM 54
Gh DNF-YB18 MERG.GFHGYRKLPDNTSGLKVT..........EMNMRMGEANHTNSHS...............NSDDNE.........CTVREQDRFM 54
Gh ANF-YB22 MERGDGFNRFYKHAKSSSGLSIM.HGDSSNSNTNTNTIIN..NNAANNG...............NAANSN...TMPPP.CMVREQDQYM 67
Gh DNF-YB22 MERGDGFNRFYKHAKSSSGMSIM.HGDSSNS..NTNTIIN..NNAANNG...............NAANSN...TMPPP.CMVREQDQYM 65
Gh ANF-YB6 PIANVIRIMRRILPPHAKISDEAKETIQECVSEFISFITGEANERCQSEQRKTVTAEDILCAMGKLGFDDYMEPLTVYLTRYRQSENERT 180
Gh DNF-YB6 PIANVIRIMRRILPPHAKISDEAKETIQECVSEFISFITGEANERCQSEQRKTVTAEDILCAMGKLGFDDYIEPLTVYLTRYRQSENERT 167
Gh ANF-YB18 PIANVIRIMRKILPPHAKISDDAKETIQECVSEYISFITGEANEHCQREQRKTITAEDVLWAMSKLGFDDYIEPLTVYLHRYRELEGERG 144
Gh DNF-YB18 PIANVIRIMRKILPPHAKISDDAKETIQECVSEYISFITGEANEHCQREQRKTITAEDVLWAMSKLGFDDYIEPLTVYLHRYRELEGERG 144
Gh ANF-YB22 PIANVIRIMRRILPTHAKISDDAKETIQECVSEYISFITGEANERCQREQRKTITAEDVLWAMGKLGFDDYVEPLTVFLNRYRENENERT 157
Gh DNF-YB22 PIANVIRIMRRILPTHAKISDDAKETIQECVSEYISFITGEANERCQREQRKTITAEDVLWAMGKLGFDDYVEPLTVFLNRYRENENERT 155
Gh ANF-YB6 SLRGDTFLKRGNAYGP.MMTPPHGVAPENAGFQEGMTDATSAAARAIMGGYNHGAPPGGAAGSSSQQ.APFDNNLDPFDVF 259
Gh DNF-YB6 SLRGDTFLKRGNAYGP.MIIPPHGVEPENAGFQEGMTDATSAAARAIMGGYNHGAHPGGAAGSSSQQ.APFDNNLDPFVEF 246
Gh ANF-YB18 SIRGEPVVKRVVDYGT.LGVAAFAPA.FHMGHHHHHGHG..FFGSGAMGGYLKDESSAGSSQAAVANGEPYAQQHK..... 216
Gh DNF-YB18 SIRGEPVVKRVVDYGT.LGVAAFAPA.FHMGHHHHHGHG..FFGSGAMGGYLKDESSAGSSQAAVANGEPYAQQHK..... 216
Gh ANF-YB22 SLRSEPMLKRGIDYGPSMMMAPYGAG.FHVGHQQGIFDG.....ATAMGGYMRDGSSGGGGEPSSQ..ASLANHFDPFGQF 230
Gh DNF-YB22 SLRSEPMLKRGIDYGPSMMMAPYGAG.FHVGHQQGIFDG.....ATAMGGYMRDGSSGGGGGPSSQ..ASLGNHFDPFGQF 228
