# Supplementary material for: Genome-Wide Analysis of the NF-YB Gene Family in Gossypium hirsutum L. and Characterization of the Role of GhDNF-YB22 in Embryogenesis
Source: Int J Mol Sci. 2018 Feb 6;19(2):483. doi: 10.3390/ijms19020483 (PMC5855705; doi:10.3390/ijms19020483)
Supplement: Supplementary file 1 [file ijms-19-00483-s001.zip › ijms-265162-supplementary/supplementary materials/Supplementary Figure S3.pdf]

```
Gh_ANF-YB4      1 ------------------MATS--APVPASPGGGG---------SHESGGEQSPR-----
Gh_DNF-YB4      1 ------------------MATS--APVPASPGGGG---------SHESGGEQSPR-----
Gh_ANF-YB16     1 -------------------MAE--APAPASPGGG----------SHESG-EQSPR-----
Gh_DNF-YB16     1 -------------------MAE--ALAPASPGGG----------SHESG-EQSPK-----
Gh_ANF-YB19     1 -------------------MADGMGGGPTSPAGG----------SHESGGEHSSPQ----
Gh_DNF-YB19     1 -------------------MADGMGGGPTSPAGG----------SHESGGEHSSPQ----
Gh_ANF-YB21     1 MDTNMFHFHFPSDLRSFKDMADGMARGPTSPAGG----------SHESG-EQCSSH----
Gh_DNF-YB21     1 -------MHFG-DLRSFKDMTDGMARGPTSPAGG----------SHESGGEQCSSH----
Gh_ANF-YB3      1 -------------------MADSDSESGGAQ--------------NNASNAAGNNNH---
Gh_DNF-YB3      1 -------------------MADSDSESGGAQ--------------NNASNAAGNNNH---
Gh_ANF-YB23     1 -------------------MVDSDTESGGGQ--------------NNASNAD--------
Gh_DNF-YB23     1 -------------------MVDSDTESGGGP--------------NNASNAD--------
Gh_ANF-YB2      1 -------------------MADSDDESGEQN--------------NNGGNAHS-------
Gh_DNF-YB2      1 -------------------MADSDDESGEQN--------------HNGGNAHS-------
Gh_ANF-YB14     1 -------------------MAESDNESGGHN--------------NSGGNAHS-------
Gh_DNF-YB14     1 -------------------MAESDNESGGHN--------------NSGGNAHS-------
Gh_ANF-YB5      1 -------------------MTGKRNQTSPVG--------------SPSSGNIS-------
Gh_DNF-YB5      1 -------------------MTGKRNQTSPVG--------------SPSSGNIS-------
Gh_ANF-YB8      1 -------------------MAENIG---------------------TSNDGGGDG-----
Gh_DNF-YB8      1 -------------------MAENIG---------------------TSNDGGGDG-----
Gh_ANF-YB10     1 -------------------MAENAG-------------------TSGTTSNNGNN-----
Gh_DNF-YB10     1 -------------------MAPNIDVQLGDQQVFKVFNNGRKCWNSGTTSNNGNS-----
Gh_ANF-YB20     1 -------------------MAENVG---------------------ASCSNDDD------
Gh_DNF-YB20     1 -------------------MAENVG---------------------GSGSNDDD------
Gh_ANF-YB7      1 -------------------MADKIGINNLDS----EGLKYNFATGAASSVLSGED-----
Gh_DNF-YB7      1 -------------------MADKIGINNLDS----EGLKYNFAAGADSSVISGED-----
Gh_ANF-YB13     1 -------------------MTDKIGIDSYR-----EGHKYDFGGGGGA---SGED-----
Gh_DNF-YB13     1 -------------------MTDKIGIDSYR-----EGHKYDFGGGGDG---SGED-----
Gh_ANF-YB15     1 -------------------MGD--------------------------------------
Gh_DNF-YB15     1 -------------------MGD--------------------------------------
Gh_ANF-YB24     1 -------------------MVD--------------------------------------
Gh_DNF-YB24     1 -------------------MVD--------------------------------------
Gh_ANF-YB9      1 -------------------MEDE----NAAHEHNKGSPESP---CAKSGGSSNN------
Gh_DNF-YB9      1 -------------------MEDE----NAAHGHNKGSPESP---CAKSGGSSNN------
Gh_ANF-YB12     1 -------------------MEDEIKHGNVTHVPNKGSPESPHVTCCDSNNSNNNNNQ---
Gh_DNF-YB12     1 -------------------MEDEIKHGNVTHVPNKGSPESPHVACSDSNHNQNN------
Gh_ANF-YB18     1 -------------MERG-GFHGYRKLPDNTSGLKVAEMNMRMGEANHTNSHSNSDD----
Gh_DNF-YB18     1 -------------MERG-GFHGYRKLPDNTSGLKVTEMNMRMGEANHTNSHSNSDD----
Gh_SNF-YB18     1 -------------------------------------MNMRMGEANHTNSHSNSDD----
Gh_ANF-YB6      1 -------------MERGDEFSRFPKLAKSNSGLGIIQHGDSSDSINNVNNIFNITDSVSN
Gh_DNF-YB6      1 -------------MERGDEFSRFPKLAKSNSGLGIIQHGDSSNSINNVNNIFNVTD----
Gh_ANF-YB22     1 -------------MERGDGFNRFYKHAKSSSGL-SIMHGDSSNSNTNTNTIINNNA----
Gh_DNF-YB22     1 -------------MERGDGFNRFYKHAKSSSGM-SIMHGDSSNS--NTNTIINNNA----
Gh_SNF-YB22     1 ------------------------------------------------------------
Gh_ANF-YB17     1 -------------------MEKVVSEAE--------------------------------
Gh_DNF-YB17     1 -------------------MEKVVSEAE--------------------------------
Gh_ANF-YB1      1 -------------------MEPMDIVGK--------------------------------
Gh_DNF-YB1      1 -------------------MEPMDIVGK--------------------------------
Gh_ANF-YB11     1 -------------------MEPMDIVGK--------------------------------
Gh_DNF-YB11     1 -------------------MEPMDIVGK--------------------------------
```

```
Gh_ANF-YB4     27 ---------------------------------SNVREQERFLPIANISRIMKKALPANA
Gh_DNF-YB4     27 ---------------------------------SNVREQERFLPIANISRIMKKALPANA
Gh_ANF-YB16    24 ---------------------------------SNVREQDRFLPIANISRIMKKALPANG
Gh_DNF-YB16    24 ---------------------------------SNVREQDRFLPIANISRIMKKALPANG
Gh_ANF-YB19    28 ---------------------------------STVREQDRYLPIANISRIMKKALPSNG
Gh_DNF-YB19    28 ---------------------------------STVREQDRYLPIANISRIMKKALPSNG
Gh_ANF-YB21    46 ---------------------------------STVREQDRYLPIANISRIMKRALPTNG
Gh_DNF-YB21    39 ---------------------------------STVREQERYLPIANISRIMKTALPTNG
Gh_ANF-YB3     25 --------------------------------LFSPKEQDRFLPIANVGRIMKKALPANA
Gh_DNF-YB3     25 --------------------------------LFSPKEQDRFLPIANVGRIMKKALPANA
Gh_ANF-YB23    20 --------------------------------LSSPKEQDRFLPIANVSRIMKKALPANA
Gh_DNF-YB23    20 --------------------------------LSSPKEQDRFLPIANVSRIMKKALPANA
Gh_ANF-YB2     21 --------------------------------EASGREQDRFLPIANVSRIMKKALPPNA
Gh_DNF-YB2     21 --------------------------------EASGREQDRFLPIANVSRIMKKALPPNA
Gh_ANF-YB14    21 --------------------------------ETSAREQDRFLPIANVSRIMKKALPANA
Gh_DNF-YB14    21 --------------------------------ETSAREQDRFLPIANVSRIMKKALPANA
Gh_ANF-YB5     21 --------------------------------DSSSKEQDRFLPIANVSRIMKKSLPANA
Gh_DNF-YB5     21 --------------------------------DSSSKEQDRFLPIANVSRIMKKSLPANA
Gh_ANF-YB8     16 ---------------------------------GGFKEQDHLLPIANVGRIMKQILPPNA
Gh_DNF-YB8     16 ---------------------------------YGFKEQDHLLPIANVGRIMKQILPPNA
Gh_ANF-YB10    18 ---------------------------------IGFKEQDQLLPIANVGRIMKQILPPNA
Gh_DNF-YB10    37 ---------------------------------VGFKEQDQLLPIANVGRIMKQILPPNA
Gh_ANF-YB20    15 ----------------------------------GFREQDRLLPIANVGRIMKQMLPPNA
Gh_DNF-YB20    15 ----------------------------------GFREQDRLLPIANVGRIMKQMLPPNA
Gh_ANF-YB7     33 ---------------------------------GIIKEQDRLLPIANVGRIMKQILPPNA
Gh_DNF-YB7     33 ---------------------------------GIIKEQDRLLPIANVGRIMKQILPPNA
Gh_ANF-YB13    29 ---------------------------------GFIKEQDRLLPIANVGRIMKQILPPNA
Gh_DNF-YB13    29 ---------------------------------GFIKEQDRLLPIANVGRIMKQILPPNA
Gh_ANF-YB15     4 -------------------------------------EQDPLLPIANVGRIMKRILPPTG
Gh_DNF-YB15     4 -------------------------------------EQNPFLPIANVGRIMKQILPPSA
Gh_ANF-YB24     4 -------------------------------------GQERLLPIANVGRIMKQILPPSA
Gh_DNF-YB24     4 -------------------------------------EQERLLPIANVGRIMKQILPPNA
Gh_ANF-YB9     29 --------------------------------NNN-KEQDRFLPIANVGRIMKKVIPSNG
Gh_DNF-YB9     29 --------------------------------NNNNKEQDRFLPIANVGRIMKKVIPSNG
Gh_ANF-YB12    39 --------------------------------NNNNKEQDRFLPIANVSRILKKVIPSNG
Gh_DNF-YB12    36 ---------------------------------NNNKEQDRFLPIANVSRILKKVIPSNG
Gh_ANF-YB18    43 -------------------------------NECTVREQDRFMPIANVIRIMRKILPPHA
Gh_DNF-YB18    43 -------------------------------NECTVREQDRFMPIANVIRIMRKILPPHA
Gh_SNF-YB18    20 -------------------------------NECTVREQDRFMPIANVIRIMRKILPPHA
Gh_ANF-YB6     48 AGNIFNITANVSNAGNISNTSNTNAVSTMPPPGPVLREQDQYMPIANVIRIMRRILPPHA
Gh_DNF-YB6     44 ---------NVSNAGNISNTSNTNAVSTMPPPGPVLREQDQYMPIANVIRIMRRILPPHA
Gh_ANF-YB22    43 -----------ANNGNAANSN------TMPPP-CMVREQDQYMPIANVIRIMRRILPTHA
Gh_DNF-YB22    41 -----------ANNGNAANSN------TMPPP-CMVREQDQYMPIANVIRIMRRILPTHA
Gh_SNF-YB22     1 ----------------------------MPPP-CMVREQDQYMPIANVIRIMRRILPTHA
Gh_ANF-YB17    10 ------------------------------------ELPKAIVRRVVKEKLSECSPDYDF
Gh_DNF-YB17    10 ------------------------------------ELPKAIVRRVVKEKLSECSPDYDF
Gh_ANF-YB1     10 ------------------------------------SKEDASLPKATMTKIIKEMLPPDV
Gh_DNF-YB1     10 ------------------------------------SKEDASLPKATMTKIIKEMLPPDV
Gh_ANF-YB11    10 ------------------------------------SKEDASLPKATMTKIIKEMLPPDV
Gh_DNF-YB11    10 ------------------------------------SKEDASLPKATMTKIIKEMLPPDV
```

```
Gh_ANF-YB4     54  KIAKDAKETVQECVSEFISFITSEASDKCQKEKRKTINGDDLLWAMATLGFEDYIDPLKI
Gh_DNF-YB4     54  KIAKDAKETVQECVSEFISFITSEASDKCQKEKRKTINGDDLLWAMATLGFEDYIDPLKI
Gh_ANF-YB16    51  KIAKDAKETVQECVSEFISFITSEASDKCQREKRKTINGDDLLWAMATLGFEDYIDPLKI
Gh_DNF-YB16    51  KIAKDAKETVQECVSEFISFITSEASDKCQREKRKTINGDDLLWAMATLGFEDYIDPLRI
Gh_ANF-YB19    55  KIAKDAKDTVQECVSEFISFITSEASDKCQKEKRKTINGDDLLWAMATLGFEDYIEPLKI
Gh_DNF-YB19    55  KIAKDAKDTVQECVSEFISFITSEASDKCQKEKRKTINGDDLLWAMATLGFEDYIEPLKI
Gh_ANF-YB21    73  KIAKDAKETVQECVSEFISFITSEASDKCQKEKRKTINGDDLLWAMATLGFEDYIEPLKI
Gh_DNF-YB21    66  KIAKDAKETVQESVSEFISFITSEASDKCQKEKRKTINGDDLLWAMATLGFEDYIEPLKI
Gh_ANF-YB3     53  KISKEAKETVQECVSEFISFITGEASDKCQKEKRKTINGDDLLWAMTTLGFEDYVEPLKV
Gh_DNF-YB3     53  KISKEAKETVQECVSEFISFITGEASDKCQKEKRKTINGDDLLWAMTTLGFEDYVEPLKV
Gh_ANF-YB23    48  KISKEAKETVQECVSEFISFITGEASDKCQKEKRKTINGDDLLWAMTTLGFEDYVEPLKV
Gh_DNF-YB23    48  KISKEAKETVQECVSEFISFITGEASDKCQKEKRKTINGDDLLWAMTTLGFEDYVEPLKV
Gh_ANF-YB2     49  KISKDAKETVQECVSEFISFITGEASDKCQREKRKTINGDDLLWAMMTLGFEEYVEPLKI
Gh_DNF-YB2     49  KISKDAKETVQECVSEFISFITGEASDKCQREKRKTINGDDLLWAMMTLGFEEYVEPLKI
Gh_ANF-YB14    49  KISKDAKETVQECVSEFISFITGEASDKCQREKRKTINGDDLLWAMTTLGFEEYVEPLKI
Gh_DNF-YB14    49  KISKDAKETVQECVSEFISFITGEASDKCQREKRKTINGDDLLWAMTTLGFEEYVEPLKI
Gh_ANF-YB5     49  KISKEAKETVQECVSEFISFITGEASDKCQREKRKTINGDDLLWAMTTLGFENYVGPLKV
Gh_DNF-YB5     49  KISKEAKETVQECVSEFISFITGEASDKCQREKRKTINGDDLLWAMTTLGFENYVGPLKV
Gh_ANF-YB8     43  KISKEAKETMQECVSEFISFVTGEASEKCRKERRKTVNGDDICWALATLGFDDYAVPLKR
Gh_DNF-YB8     43  KISKEAKETMQECVSEFISFVTGEASEKCRKERRKTVNGDDICWALATLGFDNYAVPLKR
Gh_ANF-YB10    45  KISKEAKETMQECASEFISFVTGEASEKCKKERRKTVNGDDICWALATLGLDDYAVPLKR
Gh_DNF-YB10    64  KISKEAKETMQECASEFISFVTGETSEKCKKERRKTVNGDDICWALATLGLDDYAVPLKR
Gh_ANF-YB20    41  KISKEAKETMQECVSEFISFVTSEASDKCRKERRKTINGEDICWALVTLGFDDYAAPLRR
Gh_DNF-YB20    41  KISKEAKETMQECVSEFISFVTSEASDKCRKERRKTINGEDICWALVTLGFDDYAAPLRR
Gh_ANF-YB7     60  KISKEAKETMQECVSEFISFVTGEASEKCHKEKRKTVNGDDVCWALATLGFDNYAEQLKR
Gh_DNF-YB7     60  KISKEAKETMQECVSEFISFVTGEASDKCHKEKRKTVNGDDVCWALATLGFDNYAEQLKR
Gh_ANF-YB13    56  KVSKEAKETMQECVSEFISFVTGEASDKCHREKRKTVNGDDICWALATLGFDNYAEQLKR
Gh_DNF-YB13    56  KVSKEAKETMQECVSEFISFVTGEASDKCHREKRKTVNGDDICWALATLGFDNYAEQLKR
Gh_ANF-YB15    27  KVSKEAKETMQECVTEFISFVTSDASDKCRKESRKTIYGDDICRALGAVGLDNYAEAIVR
Gh_DNF-YB15    27  KVSKEAKETMQECVTEFISFVTSEASDKCRKGSRKTICGDDICWALGAVGLDNYAQAMVR
Gh_ANF-YB24    27  KVSKEAKQTLQECATEFISFVTGEASDKCRKENRKTVNGDDICWALGALGFDNYADAIVR
Gh_DNF-YB24    27  KVSKEAKQTLQECATEFISFVTGEASDKCRKENRKTVNGDDICWALGALGFDNYADAIVR
Gh_ANF-YB9     56  KISKDAKETVQECVSEFISFVTGEASDKCQREKRKTINGDDIIWAITTLGFEEYVGPLKL
Gh_DNF-YB9     57  KISKDAKETVQECVSEFISFVTGEASDKCQREKRKTINGDDIIWAITTLGFEEYVGPLKL
Gh_ANF-YB12    67  KISKDAKETIQECVSEFISFVTGEASDKCQREKRKTINGDDIIWAITTLGFEEYVGPLKL
Gh_DNF-YB12    63  KISKDAKETIQECVSEFISFVTGEASDKCQREKRKTINGDDIIWAITTLGFEEYVGPLKL
Gh_ANF-YB18    72  KISDDAKETIQECVSEYISFITGEANEHCQREQRKTITAEDVLWAMSKLGFDDYIEPLTV
Gh_DNF-YB18    72  KISDDAKETIQECVSEYISFITGEANEHCQREQRKTITAEDVLWAMSKLGFDDYIEPLTV
Gh_SNF-YB18    49  KISDDAKETIQECVSEYISFITGEANEHCQREQRKTITAEDVLWAMSKLGFDDYIEPLTV
Gh_ANF-YB6    108  KISDEAKETIQECVSEFISFITGEANERCQSEQRKTVTAEDILCAMGKLGFDDYMEPLTV
Gh_DNF-YB6     95  KISDEAKETIQECVSEFISFITGEANERCQSEQRKTVTAEDILCAMGKLGFDDYIEPLTV
Gh_ANF-YB22    85  KISDDAKETIQECVSEYISFITGEANERCQREQRKTITAEDVLWAMGKLGFDDYVEPLTV
Gh_DNF-YB22    83  KISDDAKETIQECVSEYISFITGEANERCQREQRKTITAEDVLWAMGKLGFDDYVEPLTV
Gh_SNF-YB22    32  KISDDAKETIQECVSEYISFITGEANERCQREQRKTITAEDVLWAMGKLGFDDYVEPLT-
Gh_ANF-YB17    34  NVHKDAHLAFTESARIFIHYLSATANDICKESKRQTMNAEDVFKALEEIEFSEFVKPLRA
Gh_DNF-YB17    34  NVHKDAHLAFTESARIFIHYLSATANDICKESKRQTMNAEDVFKALEEIEFSEFVKPLGA
Gh_ANF-YB1     34  RVARDAQDLLIECCVEFINLISSESNEVCNREDKRTIAPEHVLKALEVLGFGKYIEEVYA
Gh_DNF-YB1     34  RVARDAQDLLIECCVEFINLISSESNEVCNREDKRTIAPEHVLKALEVLGFGKYIEEVYA
Gh_ANF-YB11    34  RVARDTQDLLIECCVEFINLISSESNEVCNREEKRTIAPEHVLKALEVLGFGEYIEEVYA
Gh_DNF-YB11    34  RVARDTQDLLIECCVEFINLISSESNEVCNREEKRTIAPEHVLKALEVLGFGEYIEEVYA
```

```
Gh_ANF-YB4    114 YLTKYRE--GDTKGSVKGGDTFAKKDVQ---PGPNA-----------------------Q
Gh_DNF-YB4    114 YLTKYRE--GDTKGSVKGGDTFAKKDVQ---PGPNA-----------------------Q
Gh_ANF-YB16   111 YLSRYRE--GDAKGSAKGGDASAKKDVQ---PGPNG-----------------------Q
Gh_DNF-YB16   111 YLSRYRE--GDAKGSAKGGDASAKKDVQ---PGPNG-----------------------Q
Gh_ANF-YB19   115 YLARYRE--GDTKGSARGGDGSFKRDAAGALPAQNP-----------------------Q
Gh_DNF-YB19   115 YLARYRELEGDTKGSARGGDGSFKRDAAGALPAQNP-----------------------Q
Gh_ANF-YB21   133 YLARYRE--GDAKGSIRG-EVPLKRDAVRVLAVPNP-----------------------Q
Gh_DNF-YB21   126 YLARYRELEGDAKGSVRG-EVPLKRDAVRVLSVPNP-----------------------Q
Gh_ANF-YB3    113 YLQRFREMEGEKTTVAR--DKDAPLVAASGGG-----GGGGG------------------
Gh_DNF-YB3    113 YLQRFREMEGEKTTVAR--DKDAPLVAASGGG-----GGG--------------------
Gh_ANF-YB23   108 YLQRFREMEGEKTAVAR--DKDAPLAGGVGAAAA---AGSSG------------------
Gh_DNF-YB23   108 YLQRFREMEGEKTAVAR--DKDAPLAGGVGAAAP---AGSSG------------------
Gh_ANF-YB2    109 YLLKYREMEGEKSSMGRG-EKDGASGGSSGGASG--GGGGGGSVGGGGVFGSGGGEFNGG
Gh_DNF-YB2    109 YLLKYREMEGEKSSMGRG-EKDGASGGSSGGASG--GGAAEG------------------
Gh_ANF-YB14   109 YLQKYREMEGEKSSMGRG-EKDGASGGSSGGASGSGGGGSGGGGGVGPGGGGGGGGFNGG
Gh_DNF-YB14   109 YLQKYREMEGEKSSMGRG-EKDGASGGSSGGASGSGGGGSGGGGGVGPGGGGGGG-FNGG
Gh_ANF-YB5    109 YLNKYRETEGEKNSMARQEDHHHHHHHQSPTSYGLISHGGANEFNN-----------VNA
Gh_DNF-YB5    109 YLNKYRETEGEKNSMARQEDHHHHHHHQSPTSYGLISHGGANEFNN-----------VSA
Gh_ANF-YB8    103 YLYKFREFEGDK--AANQVKVSIS-NSKDDD-----------------------------
Gh_DNF-YB8    103 YLYKFREFEGDK--AANQVKVSIS-NSKDDD-----------------------------
Gh_ANF-YB10   105 YLLRYRELEGEQKPAANHDKVAIVDNCNVED-----------------------------
Gh_DNF-YB10   124 YLLRYRELEGEHKPAANHDKVAIVDNCNVED-----------------------------
Gh_ANF-YB20   101 YLNKL--------ITT-VMKAGMIGSSRN-------------------------------
Gh_DNF-YB20   101 YLNKL--------ITTTVMKASMIGSSRN-------------------------------
Gh_ANF-YB7    120 YLHRYREQEGERVSQNRA-------IERS-------------------------------
Gh_DNF-YB7    120 YLHRYREQEGERVSQNRA-------IERS-------------------------------
Gh_ANF-YB13   116 YLQRYREQEGERANQNSAGNVHEGKEETSTYRG---------------------------
Gh_DNF-YB13   116 YLQRYREQEGERANQHSAGNVPEGKEETSTYRG---------------------------
Gh_ANF-YB15    87 HLHKYRVAALN---QHKATTSS--FEDKMKN-----------------------------
Gh_DNF-YB15    87 YLHKYRVAALN---QQKATTSS--FEDKDEES----------------------------
Gh_ANF-YB24    87 YLHKYREVERDKATQNKATCIS--SQDKDEESS---------------------------
Gh_DNF-YB24    87 YLHKYREVERDKATQNKATCIS--SQDKDEESS---------------------------
Gh_ANF-YB9    116 YLTKYREIEGEKLNLPKQQRSEQKQHQQSKHEQNIAFNTN---------VYSSTNLLS--
Gh_DNF-YB9    117 YLTKYREIEGEKLNLPKQQRSEQKQHQQSKHEQNIAFNTN---------VYSSTNLLS--
Gh_ANF-YB12   127 YLSKYREMEGEKLIFPKQQRSDQR-RQHSEYEQNIVFNNNNNININ---TNSNNNVYSSI
Gh_DNF-YB12   123 YLSKYREMEGEKLIFPKQQRSDQR-RQHSEYEQNIVFNNNNNININ---TNSNNNVYSSI
Gh_ANF-YB18   132 YLHRYRELEGERGSIRGEPVVKRVVDYGT-LGVAAFAPA-------------------FH
Gh_DNF-YB18   132 YLHRYRELEGERGSIRGEPVVKRVVDYGT-LGVAAFAPA-------------------FH
Gh_SNF-YB18   109 YLHRYRELEGERGSIRGEPVVKRVVDYGT-LGVAAFAPA-------------------FH
Gh_ANF-YB6    168 YLTRYRQSENERTSLRGDTFLKRGNAYGP-MMTPPHGVAP------------------FN
Gh_DNF-YB6    155 YLTRYRQSENERTSLRGDTFLKRGNAYGP-MIIPPHGVEP------------------FN
Gh_ANF-YB22   145 FLNRYRENENERTSLRSEPMLKRGIDYGPSMMMAPYGAG-------------------FH
Gh_DNF-YB22   143 FLNRYRENENERTSLRSEPMLKRGIDYGPSMMMAPYGAG-------------------FH
Gh_SNF-YB22       ------------------------------------------------------------
Gh_ANF-YB17    94 SLAEFRKKNAGKKGGAAKENEVKKKRKIEDSSAKNG------------------------
Gh_DNF-YB17    94 SLAEFRKKNAGKKGGAAKENEVKKKRKIEYSSAKNG------------------------
Gh_ANF-YB1     94 AYEQHKIETLQDSLKGGKWSNGAEMTEEEAVAEQQR------------------------
Gh_DNF-YB1     94 AYEQHKIETLQDSLKGGKWSNGAEMTEEEAVAEQQR------------------------
Gh_ANF-YB11    94 AYEQHKIETMHDSLKGGKWSNGAEMTEEEAAAEQQR------------------------
Gh_DNF-YB11    94 AYEQHKIETMHDSLKGGKWSNGAEMTEEEAAAEQQR------------------------
```

```
Gh_ANF-YB4   146 LAHQGSFSQGVYYGNSQSQSAHMMASDARH------------------------------
Gh_DNF-YB4   146 LVHQGSFSQGVYYGNSQSQSAHMMASDARH------------------------------
Gh_ANF-YB16  143 LVHQGSFSQGVNYGNSQ--VIFS-LFLSLLLCIM--------------------------
Gh_DNF-YB16  143 LVHQGSFSQGVSYGNSQGQVQFPGIHLIWRTDIMDGLSLIDVTSEDDCLIDSPLRDDTTP
Gh_ANF-YB19  150 FSIQGSLN----YINSQAQGQHMIIPSMQGNE----------------------------
Gh_DNF-YB19  152 FSIQGSLN----YINSQAQGQHMIIPSMQGNE----------------------------
Gh_ANF-YB21  167 FPIEGSLN----YINSQAQGHHMIVPSMQGNE----------------------------
Gh_DNF-YB21  162 GDAKGSVRG--EVPLKRDAVRVLSVPNPQVKQ----------------------------
Gh_ANF-YB3   148 --VYGMMVH-QHQGHVYGSTGFHHMGSGLGKGG----------------------PPNNL
Gh_DNF-YB3   146 --VYGMMVH-QHQGHVYGSTGFHQMGSGLGKGG----------------------PPNNL
Gh_ANF-YB23  145 --MYGMMVHQQHQGHVYGTGGFHQMGR-----G----------------------PR---
Gh_DNF-YB23  145 --MYGMMVHQQHQGHVYGSGGFHQMGQ-----G----------------------PR---
Gh_ANF-YB2   166 GGMYGGMMMGHHQGHMYSSGGFHHQM----------------------------------
Gh_DNF-YB2   148 ------------------------------------------------------------
Gh_ANF-YB14  168 GMYGGMMMMGHHQGHVYGSGGYHHQISMEKRGG----------------------TGVAV
Gh_DNF-YB14  167 GMYGGMMMMGHHQGHVYGSGGYHHQISMEKRGG----------------------TGVAA
Gh_ANF-YB5   158 GISSSANAADHFQGYNSGVGGFFSLGSHPQSYGDHGRRGIGGYGENLMAAAGGFNTSRIG
Gh_DNF-YB5   158 GISSSANAADHFQGYNSGGGGFFSLGSHPQSYG-----------ENLMAAAGGFNTSRMG
Gh_ANF-YB8   131 ---GDEAQKQQQQQSPLMFQHDWKQ-----------------------------------
Gh_DNF-YB8   131 ---DDEAQKQQQQ-SPLMFQHDWKQ-----------------------------------
Gh_ANF-YB10  136 ---GDSMG-------PFI------------------------------------------
Gh_DNF-YB10  155 ---GDNMG-------PLI------------------------------------------
Gh_ANF-YB20  121 ----SNLG-------PFD------------------------------------------
Gh_DNF-YB20  122 ----SNLG-------PFD------------------------------------------
Gh_ANF-YB7   142 ---DSSLATRPF------------------------------------------------
Gh_DNF-YB7   142 ---DSSLATRPF------------------------------------------------
Gh_ANF-YB13  149 -EMQSSLSLGRFEICKSIW-----------------------------------------
Gh_DNF-YB13  149 -ELHSSLSLGRFERCKSIW-----------------------------------------
Gh_ANF-YB15  113 ---RIEVASHLIKR-MKLQLNKS-------------------------------------
Gh_DNF-YB15  114 --DRSGEPSHQEDETTTPQVVMNIFI----------------------------------
Gh_ANF-YB24  118 -EDRSNQPPHQQAEAPSTRV----------------------------------------
Gh_DNF-YB24  118 -EDRSNQPPHQQAEAPSTRV----------------------------------------
Gh_ANF-YB9   165 -RHTSFVPSDQPFSLPFSSNNIQKQLQQQDQIDSVGYW----------------------
Gh_DNF-YB9   166 -RHTSFVPSDQPFSLPFSSNNIQKQLQQQDQIDSVGYW----------------------
Gh_ANF-YB12  183 NVYPSFVPSDQPFSLPFSSNSFQKQLQ---------------------------------
Gh_DNF-YB12  179 NVYPSFVPSDQPFSLPFSSNSFQKQLQ---------------------------------
Gh_ANF-YB18  172 MGHHHHHGHGFFGSG--AMGGYLKDESSAG----SSQ-AAVANGEPYAQQHK--------
Gh_DNF-YB18  172 MGHHHHHGHGFFGSG--AMGGYLKDESSAG----SSQ-AAVANGEPYAQQHK--------
Gh_SNF-YB18  149 MGHHHHHGHGFFGSG--AMGGYLKDESSAG----SSQ-AAVANGEPYAQQHK--------
Gh_ANF-YB6   209 AGFQEGMTDATSAAARAIMGGYNHGAPPGGAAGSSSQQAPFDNNLDPFDVFK--------
Gh_DNF-YB6   196 AGFQEGMTDATSAAARAIMGGYNHGAHPGGAAGSSSQQAPFDNNLDPFVEFK--------
Gh_ANF-YB22  186 VGHQQGIFDGATA-----MGGYMRDGSSGGGGEPSSQ-ASLANHFDPFGQFK--------
Gh_DNF-YB22  184 VGHQQGIFDGATA-----MGGYMRDGSSGGGGGPSSQ-ASLGNHFDPFGQFK--------
Gh_SNF-YB22      ------------------------------------------------------------
Gh_ANF-YB17  130 TKTKQKKEDNHKEEEQIEEEQEQDEEEDNEEEENHGSE----------------------
Gh_DNF-YB17  130 TKTKQKKEDNHKEEEQVEGEQEQDEEEDNEEEENHGSE----------------------
Gh_ANF-YB1   130 MFAEARARMNGGAVAPKQPDPDPSLES---------------------------------
Gh_DNF-YB1   130 MFAEARARMNGGAVAPKQPDPDPSLES---------------------------------
Gh_ANF-YB11  130 MFAEARARMNGGAVVPKQPESDPSLES---------------------------------
Gh_DNF-YB11  130 MFAEARARMNGGAVVPKQPESDPSLES---------------------------------
```

```
Gh_ANF-YB4          ------------------------------------------------------------
Gh_DNF-YB4          ------------------------------------------------------------
Gh_ANF-YB16         ------------------------------------------------------------
Gh_DNF-YB16     203 QFSGSFDEHGKLEGDETRGKGRYNLRKSLTWDSAFFSSAAFLEPEECTNTLESSENGEIH
Gh_ANF-YB19         ------------------------------------------------------------
Gh_DNF-YB19         ------------------------------------------------------------
Gh_ANF-YB21         ------------------------------------------------------------
Gh_DNF-YB21         ------------------------------------------------------------
Gh_ANF-YB3      183 GRPK--------------------------------------------------------
Gh_DNF-YB3      181 GRPK--------------------------------------------------------
Gh_ANF-YB23         ------------------------------------------------------------
Gh_DNF-YB23         ------------------------------------------------------------
Gh_ANF-YB2      192 -------NAAVRLR----------------------------------------------
Gh_DNF-YB2      148 --------VLVEV-----------------------------------------------
Gh_ANF-YB14     206 ANDEARGGAAVRSR----------------------------------------------
Gh_DNF-YB14     205 ANDEARGGAAVRSR----------------------------------------------
Gh_ANF-YB5      218 ENGDGNGNRTMAAHFHRVDW----------------------------------------
Gh_DNF-YB5      207 ENGDGNGNRTMAAHFHRVDW----------------------------------------
Gh_ANF-YB8          ------------------------------------------------------------
Gh_DNF-YB8          ------------------------------------------------------------
Gh_ANF-YB10         ------------------------------------------------------------
Gh_DNF-YB10         ------------------------------------------------------------
Gh_ANF-YB20         ------------------------------------------------------------
Gh_DNF-YB20         ------------------------------------------------------------
Gh_ANF-YB7          ------------------------------------------------------------
Gh_DNF-YB7          ------------------------------------------------------------
Gh_ANF-YB13         ------------------------------------------------------------
Gh_DNF-YB13         ------------------------------------------------------------
Gh_ANF-YB15         ------------------------------------------------------------
Gh_DNF-YB15         ------------------------------------------------------------
Gh_ANF-YB24         ------------------------------------------------------------
Gh_DNF-YB24         ------------------------------------------------------------
Gh_ANF-YB9          ------------------------------------------------------------
Gh_DNF-YB9          ------------------------------------------------------------
Gh_ANF-YB12         ------------------------------------------------------------
Gh_DNF-YB12         ------------------------------------------------------------
Gh_ANF-YB18         ------------------------------------------------------------
Gh_DNF-YB18         ------------------------------------------------------------
Gh_SNF-YB18         ------------------------------------------------------------
Gh_ANF-YB6          ------------------------------------------------------------
Gh_DNF-YB6          ------------------------------------------------------------
Gh_ANF-YB22         ------------------------------------------------------------
Gh_DNF-YB22         ------------------------------------------------------------
Gh_SNF-YB22         ------------------------------------------------------------
Gh_ANF-YB17         ------------------------------------------------------------
Gh_DNF-YB17         ------------------------------------------------------------
Gh_ANF-YB1          ------------------------------------------------------------
Gh_DNF-YB1          ------------------------------------------------------------
Gh_ANF-YB11         ------------------------------------------------------------
Gh_DNF-YB11         ------------------------------------------------------------
```

```
Gh_ANF-YB4          ------------------------------------------------------------
Gh_DNF-YB4          ------------------------------------------------------------
Gh_ANF-YB16         ------------------------------------------------------------
Gh_DNF-YB16     263 TLPGIQEDVDNYSDSSTMLHGEASTLRNSFVKKELETKDPKNVSSSKKLEYTNHDKVKQK
Gh_ANF-YB19         ------------------------------------------------------------
Gh_DNF-YB19         ------------------------------------------------------------
Gh_ANF-YB21         ------------------------------------------------------------
Gh_DNF-YB21         ------------------------------------------------------------
Gh_ANF-YB3          ------------------------------------------------------------
Gh_DNF-YB3          ------------------------------------------------------------
Gh_ANF-YB23         ------------------------------------------------------------
Gh_DNF-YB23         ------------------------------------------------------------
Gh_ANF-YB2          ------------------------------------------------------------
Gh_DNF-YB2          ------------------------------------------------------------
Gh_ANF-YB14         ------------------------------------------------------------
Gh_DNF-YB14         ------------------------------------------------------------
Gh_ANF-YB5          ------------------------------------------------------------
Gh_DNF-YB5          ------------------------------------------------------------
Gh_ANF-YB8          ------------------------------------------------------------
Gh_DNF-YB8          ------------------------------------------------------------
Gh_ANF-YB10         ------------------------------------------------------------
Gh_DNF-YB10         ------------------------------------------------------------
Gh_ANF-YB20         ------------------------------------------------------------
Gh_DNF-YB20         ------------------------------------------------------------
Gh_ANF-YB7          ------------------------------------------------------------
Gh_DNF-YB7          ------------------------------------------------------------
Gh_ANF-YB13         ------------------------------------------------------------
Gh_DNF-YB13         ------------------------------------------------------------
Gh_ANF-YB15         ------------------------------------------------------------
Gh_DNF-YB15         ------------------------------------------------------------
Gh_ANF-YB24         ------------------------------------------------------------
Gh_DNF-YB24         ------------------------------------------------------------
Gh_ANF-YB9          ------------------------------------------------------------
Gh_DNF-YB9          ------------------------------------------------------------
Gh_ANF-YB12         ------------------------------------------------------------
Gh_DNF-YB12         ------------------------------------------------------------
Gh_ANF-YB18         ------------------------------------------------------------
Gh_DNF-YB18         ------------------------------------------------------------
Gh_SNF-YB18         ------------------------------------------------------------
Gh_ANF-YB6          ------------------------------------------------------------
Gh_DNF-YB6          ------------------------------------------------------------
Gh_ANF-YB22         ------------------------------------------------------------
Gh_DNF-YB22         ------------------------------------------------------------
Gh_SNF-YB22         ------------------------------------------------------------
Gh_ANF-YB17         ------------------------------------------------------------
Gh_DNF-YB17         ------------------------------------------------------------
Gh_ANF-YB1          ------------------------------------------------------------
Gh_DNF-YB1          ------------------------------------------------------------
Gh_ANF-YB11         ------------------------------------------------------------
Gh_DNF-YB11         ------------------------------------------------------------
```

```
Gh_ANF-YB4       ------------------------------------------------------------
Gh_DNF-YB4       ------------------------------------------------------------
Gh_ANF-YB16      ------------------------------------------------------------
Gh_DNF-YB16  323 AARKKASLAVTVPVKTMKQAPARPQTSQSSRSEISTTSSLHKPPKGLSIVGPISATHTRR
Gh_ANF-YB19      ------------------------------------------------------------
Gh_DNF-YB19      ------------------------------------------------------------
Gh_ANF-YB21      ------------------------------------------------------------
Gh_DNF-YB21      ------------------------------------------------------------
Gh_ANF-YB3       ------------------------------------------------------------
Gh_DNF-YB3       ------------------------------------------------------------
Gh_ANF-YB23      ------------------------------------------------------------
Gh_DNF-YB23      ------------------------------------------------------------
Gh_ANF-YB2       ------------------------------------------------------------
Gh_DNF-YB2       ------------------------------------------------------------
Gh_ANF-YB14      ------------------------------------------------------------
Gh_DNF-YB14      ------------------------------------------------------------
Gh_ANF-YB5       ------------------------------------------------------------
Gh_DNF-YB5       ------------------------------------------------------------
Gh_ANF-YB8       ------------------------------------------------------------
Gh_DNF-YB8       ------------------------------------------------------------
Gh_ANF-YB10      ------------------------------------------------------------
Gh_DNF-YB10      ------------------------------------------------------------
Gh_ANF-YB20      ------------------------------------------------------------
Gh_DNF-YB20      ------------------------------------------------------------
Gh_ANF-YB7       ------------------------------------------------------------
Gh_DNF-YB7       ------------------------------------------------------------
Gh_ANF-YB13      ------------------------------------------------------------
Gh_DNF-YB13      ------------------------------------------------------------
Gh_ANF-YB15      ------------------------------------------------------------
Gh_DNF-YB15      ------------------------------------------------------------
Gh_ANF-YB24      ------------------------------------------------------------
Gh_DNF-YB24      ------------------------------------------------------------
Gh_ANF-YB9       ------------------------------------------------------------
Gh_DNF-YB9       ------------------------------------------------------------
Gh_ANF-YB12      ------------------------------------------------------------
Gh_DNF-YB12      ------------------------------------------------------------
Gh_ANF-YB18      ------------------------------------------------------------
Gh_DNF-YB18      ------------------------------------------------------------
Gh_SNF-YB18      ------------------------------------------------------------
Gh_ANF-YB6       ------------------------------------------------------------
Gh_DNF-YB6       ------------------------------------------------------------
Gh_ANF-YB22      ------------------------------------------------------------
Gh_DNF-YB22      ------------------------------------------------------------
Gh_SNF-YB22      ------------------------------------------------------------
Gh_ANF-YB17      ------------------------------------------------------------
Gh_DNF-YB17      ------------------------------------------------------------
Gh_ANF-YB1       ------------------------------------------------------------
Gh_DNF-YB1       ------------------------------------------------------------
Gh_ANF-YB11      ------------------------------------------------------------
Gh_DNF-YB11      ------------------------------------------------------------
```

```
Gh_ANF-YB4          ------------------------------------------------------------
Gh_DNF-YB4          ------------------------------------------------------------
Gh_ANF-YB16         ------------------------------------------------------------
Gh_DNF-YB16     383 ASLGGLNCKIEKDTKSVTGKGTTVLKTPRRPASSTGATKIAAEGKSRVGRSQVSTFLKSP
Gh_ANF-YB19         ------------------------------------------------------------
Gh_DNF-YB19         ------------------------------------------------------------
Gh_ANF-YB21         ------------------------------------------------------------
Gh_DNF-YB21         ------------------------------------------------------------
Gh_ANF-YB3          ------------------------------------------------------------
Gh_DNF-YB3          ------------------------------------------------------------
Gh_ANF-YB23         ------------------------------------------------------------
Gh_DNF-YB23         ------------------------------------------------------------
Gh_ANF-YB2          ------------------------------------------------------------
Gh_DNF-YB2          ------------------------------------------------------------
Gh_ANF-YB14         ------------------------------------------------------------
Gh_DNF-YB14         ------------------------------------------------------------
Gh_ANF-YB5          ------------------------------------------------------------
Gh_DNF-YB5          ------------------------------------------------------------
Gh_ANF-YB8          ------------------------------------------------------------
Gh_DNF-YB8          ------------------------------------------------------------
Gh_ANF-YB10         ------------------------------------------------------------
Gh_DNF-YB10         ------------------------------------------------------------
Gh_ANF-YB20         ------------------------------------------------------------
Gh_DNF-YB20         ------------------------------------------------------------
Gh_ANF-YB7          ------------------------------------------------------------
Gh_DNF-YB7          ------------------------------------------------------------
Gh_ANF-YB13         ------------------------------------------------------------
Gh_DNF-YB13         ------------------------------------------------------------
Gh_ANF-YB15         ------------------------------------------------------------
Gh_DNF-YB15         ------------------------------------------------------------
Gh_ANF-YB24         ------------------------------------------------------------
Gh_DNF-YB24         ------------------------------------------------------------
Gh_ANF-YB9          ------------------------------------------------------------
Gh_DNF-YB9          ------------------------------------------------------------
Gh_ANF-YB12         ------------------------------------------------------------
Gh_DNF-YB12         ------------------------------------------------------------
Gh_ANF-YB18         ------------------------------------------------------------
Gh_DNF-YB18         ------------------------------------------------------------
Gh_SNF-YB18         ------------------------------------------------------------
Gh_ANF-YB6          ------------------------------------------------------------
Gh_DNF-YB6          ------------------------------------------------------------
Gh_ANF-YB22         ------------------------------------------------------------
Gh_DNF-YB22         ------------------------------------------------------------
Gh_SNF-YB22         ------------------------------------------------------------
Gh_ANF-YB17         ------------------------------------------------------------
Gh_DNF-YB17         ------------------------------------------------------------
Gh_ANF-YB1          ------------------------------------------------------------
Gh_DNF-YB1          ------------------------------------------------------------
Gh_ANF-YB11         ------------------------------------------------------------
Gh_DNF-YB11         ------------------------------------------------------------
```

```
Gh_ANF-YB4         ------------------------------------------------------------
Gh_DNF-YB4         ------------------------------------------------------------
Gh_ANF-YB16        ------------------------------------------------------------
Gh_DNF-YB16   443  TNLNQSMSVASSYGEWSSDTSLSQSTSNKRSSIVRAGLGSGSHKVTVRNSDPEQVLDAST
Gh_ANF-YB19        ------------------------------------------------------------
Gh_DNF-YB19        ------------------------------------------------------------
Gh_ANF-YB21        ------------------------------------------------------------
Gh_DNF-YB21        ------------------------------------------------------------
Gh_ANF-YB3         ------------------------------------------------------------
Gh_DNF-YB3         ------------------------------------------------------------
Gh_ANF-YB23        ------------------------------------------------------------
Gh_DNF-YB23        ------------------------------------------------------------
Gh_ANF-YB2         ------------------------------------------------------------
Gh_DNF-YB2         ------------------------------------------------------------
Gh_ANF-YB14        ------------------------------------------------------------
Gh_DNF-YB14        ------------------------------------------------------------
Gh_ANF-YB5         ------------------------------------------------------------
Gh_DNF-YB5         ------------------------------------------------------------
Gh_ANF-YB8         ------------------------------------------------------------
Gh_DNF-YB8         ------------------------------------------------------------
Gh_ANF-YB10        ------------------------------------------------------------
Gh_DNF-YB10        ------------------------------------------------------------
Gh_ANF-YB20        ------------------------------------------------------------
Gh_DNF-YB20        ------------------------------------------------------------
Gh_ANF-YB7         ------------------------------------------------------------
Gh_DNF-YB7         ------------------------------------------------------------
Gh_ANF-YB13        ------------------------------------------------------------
Gh_DNF-YB13        ------------------------------------------------------------
Gh_ANF-YB15        ------------------------------------------------------------
Gh_DNF-YB15        ------------------------------------------------------------
Gh_ANF-YB24        ------------------------------------------------------------
Gh_DNF-YB24        ------------------------------------------------------------
Gh_ANF-YB9         ------------------------------------------------------------
Gh_DNF-YB9         ------------------------------------------------------------
Gh_ANF-YB12        ------------------------------------------------------------
Gh_DNF-YB12        ------------------------------------------------------------
Gh_ANF-YB18        ------------------------------------------------------------
Gh_DNF-YB18        ------------------------------------------------------------
Gh_SNF-YB18        ------------------------------------------------------------
Gh_ANF-YB6         ------------------------------------------------------------
Gh_DNF-YB6         ------------------------------------------------------------
Gh_ANF-YB22        ------------------------------------------------------------
Gh_DNF-YB22        ------------------------------------------------------------
Gh_SNF-YB22        ------------------------------------------------------------
Gh_ANF-YB17        ------------------------------------------------------------
Gh_DNF-YB17        ------------------------------------------------------------
Gh_ANF-YB1         ------------------------------------------------------------
Gh_DNF-YB1         ------------------------------------------------------------
Gh_ANF-YB11        ------------------------------------------------------------
Gh_DNF-YB11        ------------------------------------------------------------
```

```
Gh_ANF-YB4           ------------------------------------------------------------
Gh_DNF-YB4           ------------------------------------------------------------
Gh_ANF-YB16          ------------------------------------------------------------
Gh_DNF-YB16      503 GSEVTGSLDESTGVHSASIKPSGLRPPSPKLGYSNGVRLPRHTRTRSMDSYPSMSLILPQ
Gh_ANF-YB19          ------------------------------------------------------------
Gh_DNF-YB19          ------------------------------------------------------------
Gh_ANF-YB21          ------------------------------------------------------------
Gh_DNF-YB21          ------------------------------------------------------------
Gh_ANF-YB3           ------------------------------------------------------------
Gh_DNF-YB3           ------------------------------------------------------------
Gh_ANF-YB23          ------------------------------------------------------------
Gh_DNF-YB23          ------------------------------------------------------------
Gh_ANF-YB2           ------------------------------------------------------------
Gh_DNF-YB2           ------------------------------------------------------------
Gh_ANF-YB14          ------------------------------------------------------------
Gh_DNF-YB14          ------------------------------------------------------------
Gh_ANF-YB5           ------------------------------------------------------------
Gh_DNF-YB5           ------------------------------------------------------------
Gh_ANF-YB8           ------------------------------------------------------------
Gh_DNF-YB8           ------------------------------------------------------------
Gh_ANF-YB10          ------------------------------------------------------------
Gh_DNF-YB10          ------------------------------------------------------------
Gh_ANF-YB20          ------------------------------------------------------------
Gh_DNF-YB20          ------------------------------------------------------------
Gh_ANF-YB7           ------------------------------------------------------------
Gh_DNF-YB7           ------------------------------------------------------------
Gh_ANF-YB13          ------------------------------------------------------------
Gh_DNF-YB13          ------------------------------------------------------------
Gh_ANF-YB15          ------------------------------------------------------------
Gh_DNF-YB15          ------------------------------------------------------------
Gh_ANF-YB24          ------------------------------------------------------------
Gh_DNF-YB24          ------------------------------------------------------------
Gh_ANF-YB9           ------------------------------------------------------------
Gh_DNF-YB9           ------------------------------------------------------------
Gh_ANF-YB12          ------------------------------------------------------------
Gh_DNF-YB12          ------------------------------------------------------------
Gh_ANF-YB18          ------------------------------------------------------------
Gh_DNF-YB18          ------------------------------------------------------------
Gh_SNF-YB18          ------------------------------------------------------------
Gh_ANF-YB6           ------------------------------------------------------------
Gh_DNF-YB6           ------------------------------------------------------------
Gh_ANF-YB22          ------------------------------------------------------------
Gh_DNF-YB22          ------------------------------------------------------------
Gh_SNF-YB22          ------------------------------------------------------------
Gh_ANF-YB17          ------------------------------------------------------------
Gh_DNF-YB17          ------------------------------------------------------------
Gh_ANF-YB1           ------------------------------------------------------------
Gh_DNF-YB1           ------------------------------------------------------------
Gh_ANF-YB11          ------------------------------------------------------------
Gh_DNF-YB11          ------------------------------------------------------------
```

```
Gh_ANF-YB4           ------------------------------------------------------------
Gh_DNF-YB4           ------------------------------------------------------------
Gh_ANF-YB16          ------------------------------------------------------------
Gh_DNF-YB16      563 IGPKSNTPSAYSNKMPTRTTTTTKERLPGHTRTQSMDSYPIMPKIGPRSNSPSAYSNKIP
Gh_ANF-YB19          ------------------------------------------------------------
Gh_DNF-YB19          ------------------------------------------------------------
Gh_ANF-YB21          ------------------------------------------------------------
Gh_DNF-YB21          ------------------------------------------------------------
Gh_ANF-YB3           ------------------------------------------------------------
Gh_DNF-YB3           ------------------------------------------------------------
Gh_ANF-YB23          ------------------------------------------------------------
Gh_DNF-YB23          ------------------------------------------------------------
Gh_ANF-YB2           ------------------------------------------------------------
Gh_DNF-YB2           ------------------------------------------------------------
Gh_ANF-YB14          ------------------------------------------------------------
Gh_DNF-YB14          ------------------------------------------------------------
Gh_ANF-YB5           ------------------------------------------------------------
Gh_DNF-YB5           ------------------------------------------------------------
Gh_ANF-YB8           ------------------------------------------------------------
Gh_DNF-YB8           ------------------------------------------------------------
Gh_ANF-YB10          ------------------------------------------------------------
Gh_DNF-YB10          ------------------------------------------------------------
Gh_ANF-YB20          ------------------------------------------------------------
Gh_DNF-YB20          ------------------------------------------------------------
Gh_ANF-YB7           ------------------------------------------------------------
Gh_DNF-YB7           ------------------------------------------------------------
Gh_ANF-YB13          ------------------------------------------------------------
Gh_DNF-YB13          ------------------------------------------------------------
Gh_ANF-YB15          ------------------------------------------------------------
Gh_DNF-YB15          ------------------------------------------------------------
Gh_ANF-YB24          ------------------------------------------------------------
Gh_DNF-YB24          ------------------------------------------------------------
Gh_ANF-YB9           ------------------------------------------------------------
Gh_DNF-YB9           ------------------------------------------------------------
Gh_ANF-YB12          ------------------------------------------------------------
Gh_DNF-YB12          ------------------------------------------------------------
Gh_ANF-YB18          ------------------------------------------------------------
Gh_DNF-YB18          ------------------------------------------------------------
Gh_SNF-YB18          ------------------------------------------------------------
Gh_ANF-YB6           ------------------------------------------------------------
Gh_DNF-YB6           ------------------------------------------------------------
Gh_ANF-YB22          ------------------------------------------------------------
Gh_DNF-YB22          ------------------------------------------------------------
Gh_SNF-YB22          ------------------------------------------------------------
Gh_ANF-YB17          ------------------------------------------------------------
Gh_DNF-YB17          ------------------------------------------------------------
Gh_ANF-YB1           ------------------------------------------------------------
Gh_DNF-YB1           ------------------------------------------------------------
Gh_ANF-YB11          ------------------------------------------------------------
Gh_DNF-YB11          ------------------------------------------------------------
```

```
Gh_ANF-YB4           ------------------------------------------------------------
Gh_DNF-YB4           ------------------------------------------------------------
Gh_ANF-YB16          ------------------------------------------------------------
Gh_DNF-YB16      623 ARTTTTKERLPGHTRTRSMDSYPIMPKIGQRSNSPSAYSNTIPARTTTTKVLNASRNPKI
Gh_ANF-YB19          ------------------------------------------------------------
Gh_DNF-YB19          ------------------------------------------------------------
Gh_ANF-YB21          ------------------------------------------------------------
Gh_DNF-YB21          ------------------------------------------------------------
Gh_ANF-YB3           ------------------------------------------------------------
Gh_DNF-YB3           ------------------------------------------------------------
Gh_ANF-YB23          ------------------------------------------------------------
Gh_DNF-YB23          ------------------------------------------------------------
Gh_ANF-YB2           ------------------------------------------------------------
Gh_DNF-YB2           ------------------------------------------------------------
Gh_ANF-YB14          ------------------------------------------------------------
Gh_DNF-YB14          ------------------------------------------------------------
Gh_ANF-YB5           ------------------------------------------------------------
Gh_DNF-YB5           ------------------------------------------------------------
Gh_ANF-YB8           ------------------------------------------------------------
Gh_DNF-YB8           ------------------------------------------------------------
Gh_ANF-YB10          ------------------------------------------------------------
Gh_DNF-YB10          ------------------------------------------------------------
Gh_ANF-YB20          ------------------------------------------------------------
Gh_DNF-YB20          ------------------------------------------------------------
Gh_ANF-YB7           ------------------------------------------------------------
Gh_DNF-YB7           ------------------------------------------------------------
Gh_ANF-YB13          ------------------------------------------------------------
Gh_DNF-YB13          ------------------------------------------------------------
Gh_ANF-YB15          ------------------------------------------------------------
Gh_DNF-YB15          ------------------------------------------------------------
Gh_ANF-YB24          ------------------------------------------------------------
Gh_DNF-YB24          ------------------------------------------------------------
Gh_ANF-YB9           ------------------------------------------------------------
Gh_DNF-YB9           ------------------------------------------------------------
Gh_ANF-YB12          ------------------------------------------------------------
Gh_DNF-YB12          ------------------------------------------------------------
Gh_ANF-YB18          ------------------------------------------------------------
Gh_DNF-YB18          ------------------------------------------------------------
Gh_SNF-YB18          ------------------------------------------------------------
Gh_ANF-YB6           ------------------------------------------------------------
Gh_DNF-YB6           ------------------------------------------------------------
Gh_ANF-YB22          ------------------------------------------------------------
Gh_DNF-YB22          ------------------------------------------------------------
Gh_SNF-YB22          ------------------------------------------------------------
Gh_ANF-YB17          ------------------------------------------------------------
Gh_DNF-YB17          ------------------------------------------------------------
Gh_ANF-YB1           ------------------------------------------------------------
Gh_DNF-YB1           ------------------------------------------------------------
Gh_ANF-YB11          ------------------------------------------------------------
Gh_DNF-YB11          ------------------------------------------------------------
```

```
Gh_ANF-YB4          ------------------------------------------------------------
Gh_DNF-YB4          ------------------------------------------------------------
Gh_ANF-YB16         ------------------------------------------------------------
Gh_DNF-YB16     683 KTGIIYPKHQNKSSPKPRKGSYSKAQGIGSAEKIASPDVPKLVVKLGGKGAQIKDTKIVP
Gh_ANF-YB19         ------------------------------------------------------------
Gh_DNF-YB19         ------------------------------------------------------------
Gh_ANF-YB21         ------------------------------------------------------------
Gh_DNF-YB21         ------------------------------------------------------------
Gh_ANF-YB3          ------------------------------------------------------------
Gh_DNF-YB3          ------------------------------------------------------------
Gh_ANF-YB23         ------------------------------------------------------------
Gh_DNF-YB23         ------------------------------------------------------------
Gh_ANF-YB2          ------------------------------------------------------------
Gh_DNF-YB2          ------------------------------------------------------------
Gh_ANF-YB14         ------------------------------------------------------------
Gh_DNF-YB14         ------------------------------------------------------------
Gh_ANF-YB5          ------------------------------------------------------------
Gh_DNF-YB5          ------------------------------------------------------------
Gh_ANF-YB8          ------------------------------------------------------------
Gh_DNF-YB8          ------------------------------------------------------------
Gh_ANF-YB10         ------------------------------------------------------------
Gh_DNF-YB10         ------------------------------------------------------------
Gh_ANF-YB20         ------------------------------------------------------------
Gh_DNF-YB20         ------------------------------------------------------------
Gh_ANF-YB7          ------------------------------------------------------------
Gh_DNF-YB7          ------------------------------------------------------------
Gh_ANF-YB13         ------------------------------------------------------------
Gh_DNF-YB13         ------------------------------------------------------------
Gh_ANF-YB15         ------------------------------------------------------------
Gh_DNF-YB15         ------------------------------------------------------------
Gh_ANF-YB24         ------------------------------------------------------------
Gh_DNF-YB24         ------------------------------------------------------------
Gh_ANF-YB9          ------------------------------------------------------------
Gh_DNF-YB9          ------------------------------------------------------------
Gh_ANF-YB12         ------------------------------------------------------------
Gh_DNF-YB12         ------------------------------------------------------------
Gh_ANF-YB18         ------------------------------------------------------------
Gh_DNF-YB18         ------------------------------------------------------------
Gh_SNF-YB18         ------------------------------------------------------------
Gh_ANF-YB6          ------------------------------------------------------------
Gh_DNF-YB6          ------------------------------------------------------------
Gh_ANF-YB22         ------------------------------------------------------------
Gh_DNF-YB22         ------------------------------------------------------------
Gh_SNF-YB22         ------------------------------------------------------------
Gh_ANF-YB17         ------------------------------------------------------------
Gh_DNF-YB17         ------------------------------------------------------------
Gh_ANF-YB1          ------------------------------------------------------------
Gh_DNF-YB1          ------------------------------------------------------------
Gh_ANF-YB11         ------------------------------------------------------------
Gh_DNF-YB11         ------------------------------------------------------------
```

```
Gh_ANF-YB4          ----
Gh_DNF-YB4          ----
Gh_ANF-YB16         ----
Gh_DNF-YB16   743 LGGT
Gh_ANF-YB19         ----
Gh_DNF-YB19         ----
Gh_ANF-YB21         ----
Gh_DNF-YB21         ----
Gh_ANF-YB3          ----
Gh_DNF-YB3          ----
Gh_ANF-YB23         ----
Gh_DNF-YB23         ----
Gh_ANF-YB2          ----
Gh_DNF-YB2          ----
Gh_ANF-YB14         ----
Gh_DNF-YB14         ----
Gh_ANF-YB5          ----
Gh_DNF-YB5          ----
Gh_ANF-YB8          ----
Gh_DNF-YB8          ----
Gh_ANF-YB10         ----
Gh_DNF-YB10         ----
Gh_ANF-YB20         ----
Gh_DNF-YB20         ----
Gh_ANF-YB7          ----
Gh_DNF-YB7          ----
Gh_ANF-YB13         ----
Gh_DNF-YB13         ----
Gh_ANF-YB15         ----
Gh_DNF-YB15         ----
Gh_ANF-YB24         ----
Gh_DNF-YB24         ----
Gh_ANF-YB9          ----
Gh_DNF-YB9          ----
Gh_ANF-YB12         ----
Gh_DNF-YB12         ----
Gh_ANF-YB18         ----
Gh_DNF-YB18         ----
Gh_SNF-YB18         ----
Gh_ANF-YB6          ----
Gh_DNF-YB6          ----
Gh_ANF-YB22         ----
Gh_DNF-YB22         ----
Gh_SNF-YB22         ----
Gh_ANF-YB17         ----
Gh_DNF-YB17         ----
Gh_ANF-YB1          ----
Gh_DNF-YB1          ----
Gh_ANF-YB11         ----
Gh_DNF-YB11         ----
```
