# Supplementary material for: Genome-Wide Analysis of the NF-YB Gene Family in Gossypium hirsutum L. and Characterization of the Role of GhDNF-YB22 in Embryogenesis
Source: Int J Mol Sci. 2018 Feb 6;19(2):483. doi: 10.3390/ijms19020483 (PMC5855705; doi:10.3390/ijms19020483)
Supplement: Supplementary file 1 [file ijms-19-00483-s001.zip › ijms-265162-supplementary/supplementary materials/Supplementary materials.pdf]

Table S1. Information for the genes used in the present study;
Table S2. Sequences of the primer pairs used in present study;
Table S3. Information regarding duplicated genes;
Table S4. Analysis of repeat sequences in regions 2000 bp upstream to 2000 bp downstream of *NF-YB* genes;
Table S5. Analysis of repeat sequences in regions 10,000 bp upstream to 10,000 bp downstream of *NF-YB* genes;
Figure S1. Full- length multiple alignment for GhA/DNF-YB6, 18, and 22. Residues shaded in black, pink, and green indicate 100%, >75%, and 50% similarity, respectively. The red box indicates the B domain, while yellow boxes highlight 16 conserved amino acids;
Figure S2. Chromosomal distribution of *NF-YB* genes in the *Gossypium hirsutum* genome;
Figure S3. Multiple sequence alignment of GhNF-YB proteins;
Figure S4. The results of DNA test by kanamycin (a); and the expression of *GhDNF-YB22* in different transgenic lines (b).
