# Supplementary material for: Genome-Wide Analysis of the NF-YB Gene Family in Gossypium hirsutum L. and Characterization of the Role of GhDNF-YB22 in Embryogenesis
Source: Int J Mol Sci. 2018 Feb 6;19(2):483. doi: 10.3390/ijms19020483 (PMC5855705; doi:10.3390/ijms19020483)
Supplement: Supplementary file 1 [file ijms-19-00483-s001.zip › ijms-265162-supplementary/supplementary materials/Supplementary Table S2.pdf]

Supplementary table S2: Sequences of the primer pairs used in present study

Gene name	sequence (5'-3')
GhANF-YB16	F: ATGGCGGAAGCACCGGCA
	R: CTTGAGAATTCCCATAATTA
	F: TCAAGACTGATTTGCGTTTCCA
GhHistone3	R: GCGCAAAGGTTGGTGTCTTC
	F: CGAACAATAGCACCTGAGCAT
Gh A/DNF-YB1	R: GCCTCGGCAAACATTCTTT
	F: CGTGCGAGAACAAGAGAGGT
Gh A/DNF-YB4	R: GCTGATGAACTCCGAAACG
	F: CAGACGAAGCCAAAGAAACC
Gh A/DNF-YB6	R: CGGGTCAAGTAAACGGTGAG
	F: AAGGCTTTAGAGGTTCTTGGG
Gh A/DNF-YB11	R: CATTCTTGCTCGTGCTTCA
	F: CAACCATACGAACAGCCACT
Gh A/DNF-YB18	R: TCGGACACACATTCTTGGA
	F: CGTGGTGATGGGTTCAATC
Gh A/DNF-YB22	R: GGTGTTGGTGTTGGAGTTTG
	F:CGTTGACACTTATCGGAAGC
Gh A/DNF-YB2	R:GCTTCCGATAAGTGTCAACG
	F:TATCTTGGCGTTAGCAGGC
Gh A/DNF-YB3	R:CGCAGCAGGCAACAATAA
	F:CCAAAGAAGCCAAAGAGACA
Gh A/DNF-YB5	R:GACACTTATCGGAAGCCTCG
	F:AGGAATGCGTGTCGGAGTT
Gh A/DNF-YB7	R:TCGGCGTAGTTGTCAAACC
	F:GAGCAGGACCATTTACTCCC
Gh A/DNF-YB8	R:TGAACTCCGATACGCATTCT
	F:TAGGCTTGGACGATTACGC
Gh A/DNF-YB9	R:TCAACAATGGCGACCTTATC
	F:GTAACAGAGAGGAGAAGCGGA
Gh A/DNF-YB10	R:GCTCATAAGCAGCGTAGACCT
	F:TGTGCCTAACAAAGGAAGCC
Gh A/DNF-YB12	R:TGGGAAGGAAACGGTCTTG
	F:GCAAAGGTGTCCAAAGAAGC
Gh A/DNF-YB13	R:GCCCAACAAATGTCATCCC
Gh A/DNF-YB14	F:GGCTGAATCCGACAATGAA<br>R:CGTTTCTTTAGCGTCTTTGGAG
	F:TGGGTCGGATTATGAAACG

Gh A/DNF-YB15	R:AGTTGTCAAGCCCTACAGCA
Gh A/DNF-YB17	F:GCTCGCATCTTCATCCATT
	R:CTCCGCATTCATTGTTTGC
Gh A/DNF-YB16	AAGGCTCTTCCTGCTAACGG
	CTCAAACCCTAAAGTCGCCATA
Gh A/DNF-YB19	F:AGTCAACTGTGAGGGAACAAGA
	R:GCTGATGAACTCGGAAACAC
Gh A/DNF-YB20	F:CGACGATGATGGTTTCAGG
	R:TCTTTCGCCTTTCCTTTCTG
Gh A/DNF-YB21A	F:GAGAGGGTGATGCTAAGGGA
	R:CGATAGGAAACTGTGGGTTG
Gh A/DNF-YB21D	F:GATTTGTTGTGGGCAATGG
	R:CCTTAGCATCACCCTCCAA
Gh A/DNF-YB23	F:AACGCATCCAACGCAGAT
	R:AACTCCGACACGCATTCCT
Gh A/DNF-YB24	F:CGTGGGCAGGATTATGAAA
	R:CGATTCTCTTTGCGACACTT
NPTII	F:CTATGACTGGGCACAACAGACAAT
	R:TCGTCAAGAAGGCGATAGAAGG
Gh DNF-YB22	F:TGGCGATTCTTCAAACTCC
	R:TACTCCGAGACGCATTCCT
