# Supplementary material for: Genome-Wide Analysis of the NF-YB Gene Family in Gossypium hirsutum L. and Characterization of the Role of GhDNF-YB22 in Embryogenesis
Source: Int J Mol Sci. 2018 Feb 6;19(2):483. doi: 10.3390/ijms19020483 (PMC5855705; doi:10.3390/ijms19020483)
Supplement: Supplementary file 1 [file ijms-19-00483-s001.zip › ijms-265162-supplementary/supplementary materials/Supplementary Table S3.pdf]

Supplementary table S3: Information regarding duplicated genes

| Gene | Gene type | chromosome | Gene | Gene type | chromosome |
|---|---|---|---|---|---|
| Gh_ANF-YB3 | 0 | A01 | Gh_DNF-YB3 | 4 | D01 |
| Gh_ANF-YB4 | 0 | A01 | Gh_DNF-YB4 | 1 | D01 |
| Gh_ANF-YB8 | 1 | A02 | Gh_DNF-YB8 | 1 | D02 |
| Gh_ANF-YB5 | 0 | A02 | Gh_DNF-YB5 | 1 | D03 |
| Gh_ANF-YB17 | 0 | A05 | Gh_DNF-YB17 | 0 | D05 |
| Gh_ANF-YB18 | 0 | A05 | Gh_DNF-YB18 | 0 | D05 |
| Gh_ANF-YB19 | 4 | A05 | Gh_DNF-YB19 | 4 | D05 |
| Gh_ANF-YB1 | 4 | A07 | Gh_DNF-YB1 | 4 | D07 |
| Gh_ANF-YB2 | 4 | A07 | Gh_DNF-YB2 | 4 | D07 |
| Gh_ANF-YB6 | 4 | A08 | Gh_DNF-YB6 | 1 | D08 |
| Gh_ANF-YB7 | 0 | A08 | Gh_DNF-YB7 | 0 | D08 |
| Gh_ANF-YB9 | 0 | A09 | Gh_DNF-YB9 | 0 | D09 |
| Gh_ANF-YB10 | 4 | A09 | Gh_DNF-YB10 | 4 | D09 |
| Gh_ANF-YB11 | 4 | A09 | Gh_DNF-YB11 | 4 | D09 |
| Gh_ANF-YB20 | 4 | A10 | Gh_DNF-YB20 | 4 | D10 |
| Gh_ANF-YB21 | 4 | A10 | Gh_DNF-YB21 | 4 | D10 |
| Gh_ANF-YB12 | 0 | A11 | Gh_DNF-YB12 | 0 | D11 |
| Gh_ANF-YB13 | 1 | A11 | Gh_DNF-YB13 | 1 | D11 |
| Gh_ANF-YB14 | 4 | A11 | Gh_DNF-YB14 | 4 | D11 |
| Gh_ANF-YB15 | 0 | A11 | Gh_DNF-YB15 | 0 | D11 |
| Gh_ANF-YB16 | 0 | A11 | Gh_DNF-YB16 | 0 | D11 |
| Gh_ANF-YB22 | 0 | A13 | Gh_DNF-YB22 | 0 | D13 |
| Gh_ANF-YB23 | 4 | A13 | Gh_DNF-YB23 | 4 | D13 |
| Gh_ANF-YB24 | 0 | A13 | Gh_DNF-YB24 | 0 | D13 |

Note: 0, 1, 2, 3, 4 stand for singleton, dispersed, proximal, tandem, segmental.
