# Supplementary figures and images for: Genome-Wide Analysis of the NF-YB Gene Family in Gossypium hirsutum L. and Characterization of the Role of GhDNF-YB22 in Embryogenesis
Source: Int J Mol Sci. 2018 Feb 6;19(2):483. doi: 10.3390/ijms19020483 (PMC5855705; doi:10.3390/ijms19020483)

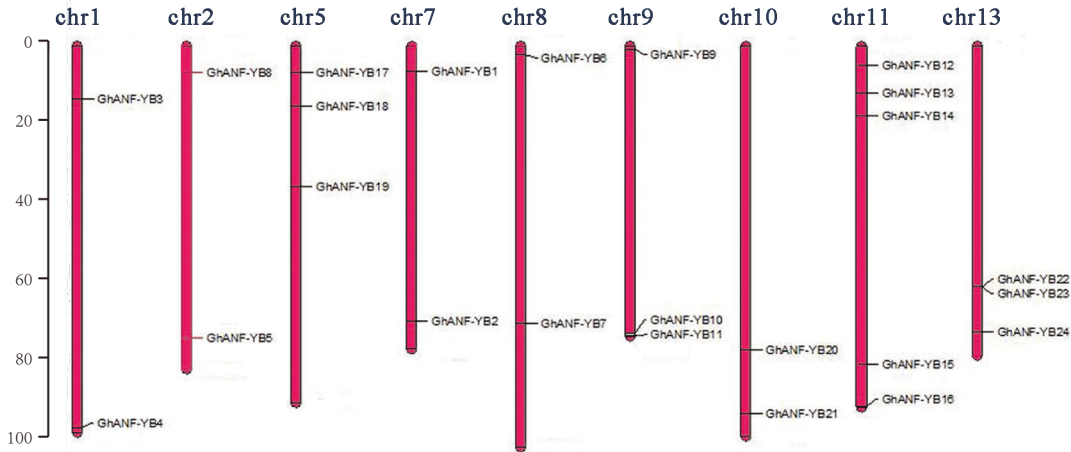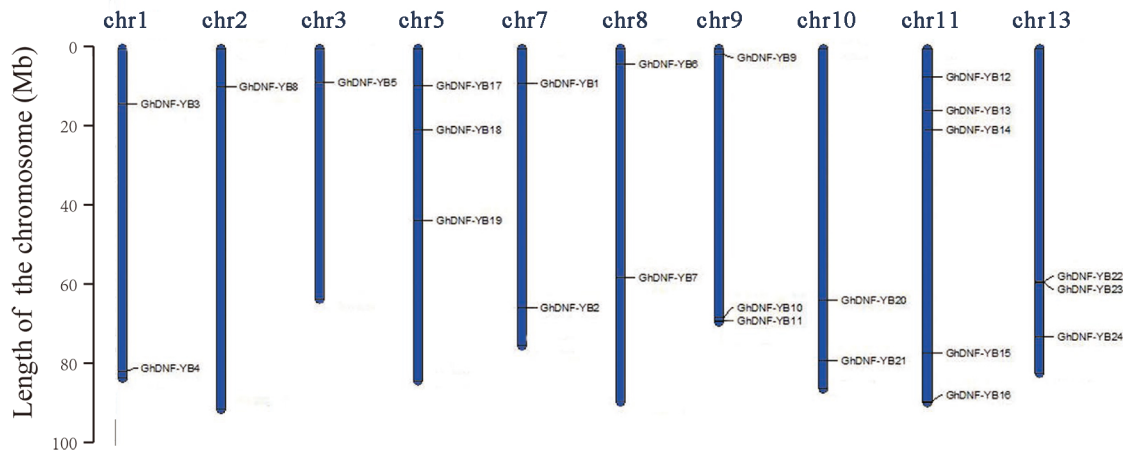

Supplement: Supplementary file 1 [file ijms-19-00483-s001.zip › ijms-265162-supplementary/supplementary materials/Supplementary Figure S2.pdf]

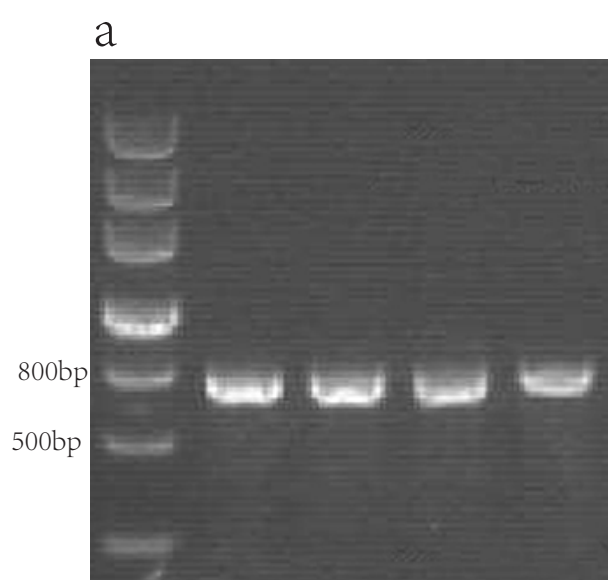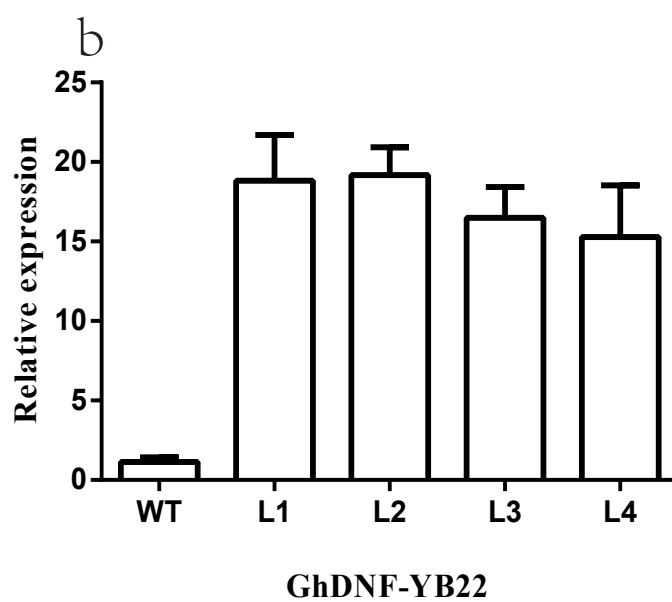

Supplement: Supplementary file 1 [file ijms-19-00483-s001.zip › ijms-265162-supplementary/supplementary materials/Supplementary Figure S4.pdf]
